# Artesunate improves venetoclax plus cytarabine AML cell targeting by regulating the Noxa/Bim/Mcl-1/p-Chk1 axis

**DOI:** 10.1038/s41419-022-04810-z

**Published:** 2022-04-20

**Authors:** Jingyi Zhang, Yuetong Wang, Chujie Yin, Ping Gong, Zhenwei Zhang, Linxiang Zhao, Samuel Waxman, Yongkui Jing

**Affiliations:** 1 Liaoning Key Lab of Targeting Drugs for Hematological Malignancies, Department of Pharmacology, Shenyang Pharmaceutical University, Shenyang, 110016 P. R. China; 2 Department of Medicinal Chemistry, Shenyang Pharmaceutical University, Shenyang, 110016 P. R. China; 3 The Division of Hematology/Oncology, Department of Medicine, The Tisch Cancer Institute, Icahn School of Medicine at Mount Sinai, New York, USA

**Keywords:** Acute myeloid leukaemia, Pharmacodynamics

## Abstract

Venetoclax plus cytarabine therapy is approved for elderly acute myeloid leukemia (AML) patients and needs further improvement. We studied the mechanisms of venetoclax plus cytarabine treatment and searched for a third agent to enhance their effects. Cytarabine induces S phase arrest-mediated DNA damage with activation of DNA replication checkpoint kinase 1 (Chk1) through phosphorylation, while venetoclax induces B cell lymphoma 2 (Bcl-2)-interacting mediator of cell death (Bim)-mediated apoptotic DNA damage. Myeloid cell leukemia-1 (Mcl-1) plays negative roles in both events by sequestering Bim and accelerating Chk1 phosphorylation. Venetoclax releases Bim from Bcl-2 with increased Bim binding to Mcl-1. Artesunate, an antimalaria drug, induces Noxa to replace Bim from Mcl-1 and induces synergistic apoptosis with venetoclax accompanied with Mcl-1 reduction. Silencing Mcl-1 or adding venetoclax/artesunate diminishes the cytarabine resistance pathway p-Chk1. The triple combination exhibits S phase arrest with enhanced DNA damage, improves AML colony formation inhibition, and prolongs survival of two mice xenograft models compared to the venetoclax/cytarabine dual combination. Artesunate serves as a bridge for venetoclax and cytarabine combination by Noxa and Bim-mediated apoptosis and Mcl-1 reduction. We provide a new triple combination for AML treatment by targeting the Noxa/Mcl-1/Bim axis to reverse Mcl-1/p-Chk1 resistance of cytarabine therapy.

## Introduction

Acute myeloid leukemia (AML) is a fatal disease treated with the typical intensive chemotherapy (cytarabine with anthracyclines) for more than 40 years and only less than 40% of patients survive for more than 5 years. This intensive therapy could not be tolerated by elderly patients who were usually treated with low-dose cytarabine alone [1]. AML patients treated by the intensive therapy were classified into poor and favorable prognosis groups based on molecular and genetic alterations [2]. Some genetic alterations have been used to develop targeted therapeutics [3]. These targeting agents such as Fms-like tyrosine kinase 3 (FLT3) inhibitors and isocitrate dehydrogenase (IDH) inhibitors only exhibit a transient efficacy and need to combine with the intensive therapy [4, 5].

AML is a disease derived from myeloid cells with blockage of differentiation and apoptosis [6]. Apoptosis is a homeostatic program of mature myeloid cells initiated through the mitochondrial apoptotic pathway [7]. The mitochondrial pathway is controlled by three types of proteins: antiapoptotic proteins Bcl-2, Bcl-xL, and Mcl-1; BH3-only proapoptotic proteins Bim, Bid, Bad, Puma, and Noxa; and proapoptotic proteins Bak and Bax. The BH3-only proteins usually bind to antiapoptotic proteins to prohibit them from activating proapoptotic proteins. Among these BH3-only proteins, Bim has multiple functions to bind all antiapoptotic proteins and to directly activate Bax/Bak, while Noxa only binds to Mcl-1 [8, 9]. Compared to normal myeloid cells, AML cells have an increased threshold of apoptosis due to upregulated antiapoptotic proteins and repressed BH3-only proteins Bim and Noxa [10]. AML cells express high levels of Bcl-2 and Mcl-1, and the levels of Mcl-1 are further increased in resistant/relapsed AML cells [11]. Several drugs used for AML therapy such as sorafenib and arsenic trioxide have been found either to down-regulate Mcl-1 or to induce Bim [12, 13]. Small molecule inhibitors of the Bcl-2 family are being developed for releasing the BH3-only proteins from the antiapoptotic proteins [14]. These inhibitors have been narrowed down from a pan-inhibitor to a specific inhibitor [15]. Venetoclax is the only Bcl-2 family inhibitor approved for leukemia therapy [16]. Venetoclax is a specific inhibitor of Bcl-2 and induces apoptosis through an Bim-dependent pathway. The high expression or induced levels of Mcl-1 have been found to cause venetoclax resistance [17]. Venetoclax alone has modest efficacy in AML patients [18], but it significantly improves the therapeutic efficacies of demethylating agents and low-dose cytarabine in elderly AML patients unfit for intensive chemotherapy [19]. The mechanisms of these venetoclax based combinations have not been solved and these combination therapies need to be further improved [19]. Considering the less toxicity and widely use of cytarabine in AML patients, we explored the mechanisms of venetoclax and cytarabine combination by focusing Bim-mediated apoptosis, DNA damage and Mcl-1 regulation. Mcl-1 is stabilized by Bim and destabilized by Noxa [20, 21]. Previously we found that dihydroartemisinin (DHA) enhances ABT-737 induced apoptosis in AML cells by inducing Noxa [22]. For a translational purpose, in this manuscript we tested artesunate, a clinical used antimalaria artemisinin, to improve venetoclax/cytarabine combination therapy by Noxa-mediated Mcl-1 reduction pathway.

## Results

### Cytarabine arrests cells in S phase and induces DNA damage with increased DNA replication checkpoint kinase 1 (Chk1) phosphorylation

**Fig. 1:**
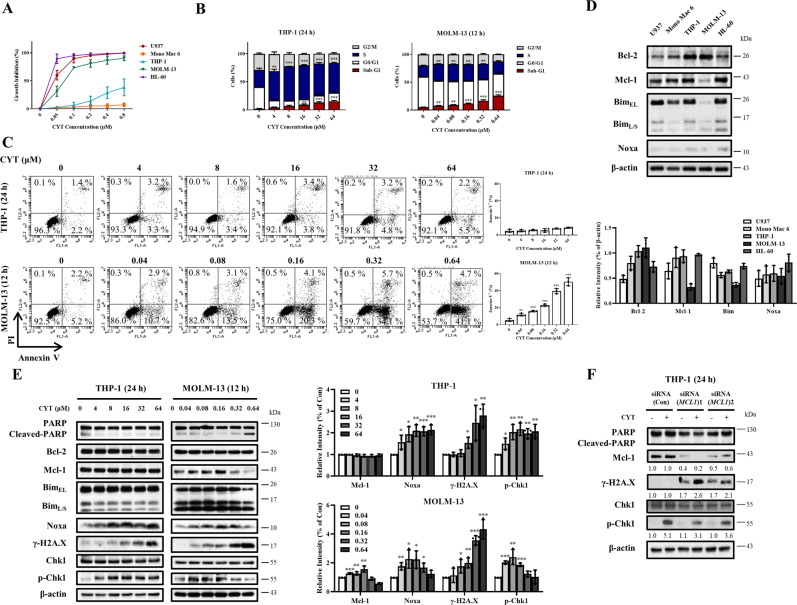
Mcl-1 levels influence cytarabine (CYT)-induced growth inhibition and DNA damage in AML cells. **A** Five AML cell lines were treated with 0.05–0.8 μM cytarabine for 72 h. Cell growth inhibition was measured by counting cell number and compared to the untreated group. **B** THP-1 and MOLM-13 cells were treated with cytarabine at the indicated concentrations and times. Cell cycle distribution assessed by FACS after PI staining. **C** Apoptotic cells determined by FACS with staining of Annexin V/PI. **D** Basal levels of Mcl-1, Bcl-2, Bim, and Noxa in five AML cell lines. **E** Relative levels of the indicated proteins of THP-1 and MOLM-13 cells after treatment with cytarabine determined by Western blotting. **F** THP-1 cells were transfected with two pairs of *MCL1* siRNAs for 24 h, then treated with 8 μM cytarabine for 24 h, and the levels of γ-H2A.X and p-Chk1 were determined by Western blotting. The column graphs are the mean ± SD of three independent experiments. *P < 0.05; **P < 0.01; ***P < 0.001 comparing to the control group by *t*-test.

U937, Mono Mac 6, THP-1, MOLM-13, and HL-60 cells were used to test the growth inhibition of cytarabine after 72 h treatment. HL-60, MOLM-13 and U937 cells were sensitive to lower concentrations of cytarabine with the concentration of inhibiting 50% cell growth (GI_50_) of 0.018 ± 0.001, 0.073 ± 0.003 and 0.021 ± 0.001 μM, respectively. THP-1 and Mono Mac 6 cells were insensitive to cytarabine with a GI_50_ of 2.6 ± 0.1 and >10 μM, respectively (Fig. 1A). We selected two cell lines, insensitive THP-1 cells and sensitive MOLM-13 cells, to compare cell cycle changes and apoptosis induction in short time treatment. THP-1 cells were treated with high concentrations (4–64 μM) for 24 h, while MOLM-13 cells were treated with lower concentrations (0.04–0.64 μM) for 12 h. The concentrations and time points selected for both cell lines were to keep cells whole and viable at the testing points. Cytarabine arrested cells in S phase and increased the cell number in SubG1 phase in both cell lines. Cytarabine is more effective to arrest cells in S phase in THP-1 cells while it is more evident to increase SubG1 phase cells in MOLM-13 cells (Fig. 1B). Based on Annexin V staining/PI assay cytarabine-induced apoptosis in MOLM-13 cells, but not in THP-1 cells (Fig. 1C). Consistent with the reported data [23, 24], cytarabine exhibits dual effects of arresting cells in S phase and inducing DNA damage. The high sensitivity of MOLM-13 cells to cytarabine is associated with DNA fragmentation and apoptosis induction. Mcl-1 has dual effects of blocking apoptosis and DNA damage. We compared the levels of Mcl-1 and its related proteins in the five AML cell lines and found that MOLM-13 cells contain lower levels of Mcl-1 and Bim than THP-1 cells (Fig. 1D). The activation of replication stress kinase Chk1 through phosphorylation has been reported to account for resistance to cytarabine therapy [25, 26] and that Mcl-1 has been reported to protect against DNA damage by accelerating Chk1 phosphorylation [27–29]. We compared the protein levels of the Mcl-1, DNA damage marker γ-H2A.X, and p-Chk1 in THP-1 and MOLM-13 cells treated with cytarabine. In THP-1 cells, cytarabine increased the levels of Noxa, p-Chk1 and γ-H2A.X, and kept Mcl-1 levels without PARP cleavage. MOLM-13 cells contain lower basal levels of Mcl-1 and Bim. To observe the protein level changes after cytarabine treatment, we took longer exposure in MOLM-13 Western blot analysis. In MOLM-13 cells, cytarabine increased the levels of Noxa, Mcl-1, and p-Chk1 at lower concentrations without PARP cleavage, but decreased the levels of Mcl-1 and p-Chk1 at increased concentrations associating with induction of γ-H2A.X and PARP cleavage. The levels of Chk1 were not changed in both cell lines after cytarabine treatment. The change of p-Chk1 levels by cytarabine treatment was associated with the level changes of Mcl-1 protein in both cell lines (Fig. 1E). Silencing Mcl-1 with siRNA in THP-1 cells attenuated p-Chk1 with enhanced γ-H2A.X of cytarabine treatment (Fig. 1F). These data suggest that the levels of Mcl-1 influence cell sensitivity to cytarabine-induced DNA damage.

### Artesunate and venetoclax synergistically induce apoptosis in AML cells

**Fig. 2:**
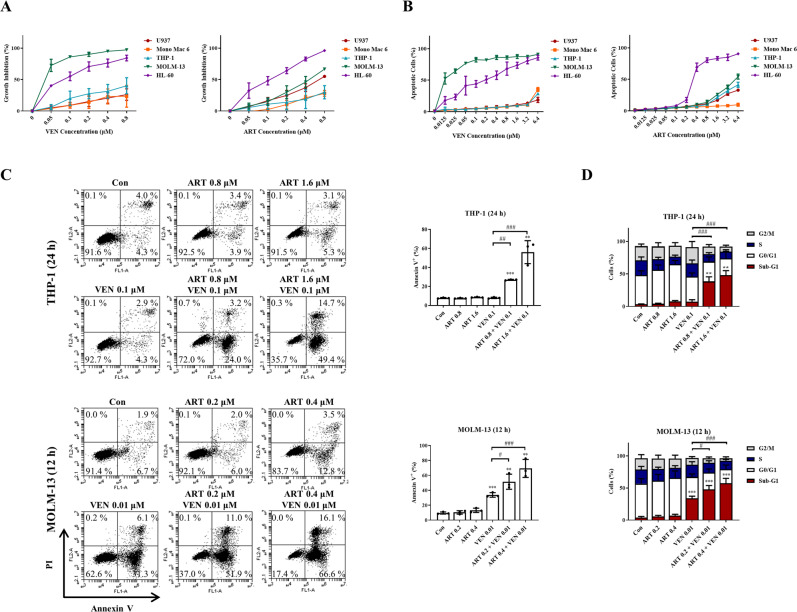
Venetoclax (VEN) in combination with artesunate (ART) induce synergistic antileukemia effects in AML cells. **A** Growth inhibition of venetoclax or artesunate in five AML cell lines. Cells were treated with venetoclax or artesunate for 72 h. Cell growth inhibition was measured by counting cell number and compared to the untreated group. **B** Apoptotic cells were determined based on morphological observation using a fluorescence microscope after staining with AO and EB. Cells were treated with venetoclax or artesunate at the indicated concentrations and times. **C** THP-1 and MOLM-13 cells were treated with venetoclax, artesunate and in combination at the indicated concentrations and times. Apoptotic cells determined by FACS after staining with Annexin V/PI. **D** Cell cycle distribution assessed by FACS after PI staining. Values are mean ± SD of three independent experiments. **P < 0.01; ***P < 0.001 compared to the control group by *t*-test. ^#^*P* < 0.05; ^##^*P* < 0.01; ^###^*P* < 0.001 by two-way ANOVA test.

The growth inhibition abilities of artesunate and venetoclax were measured in the five AML cell lines after treatment for 72 h. MOLM-13 and HL-60 cells were more sensitive to venetoclax than U937, Mono Mac 6, and THP-1 cells (GI_50_ values: U937 6.9 ± 1.2 μM, Mono Mac 6 3.2 ± 0.3 μM, THP-1 2.3 ± 0.3 μM, HL-60 0.045 ± 0.004 μM, and MOLM-13 0.0063 ± 0.0004 μM). HL-60 cells were more sensitive to artesunate than the other four cell lines (GI_50_ values: U937 0.60 ± 0.13 μM, Mono Mac 6 1.2 ± 0.2 μM, THP-1 1.8 ± 0.2 μM, HL-60 0.099 ± 0.010 μM, and MOLM-13 0.49 ± 0.01 μM) (Fig. 2A). The apoptosis induction abilities of both drugs were measured in those cell lines treated for 24 h. Similar to cell growth inhibition, MOLM-13 and HL-60 cell lines were sensitive to venetoclax, while only HL-60 cells were sensitive to artesunate (Fig. 2B). These data suggest that growth inhibition abilities of both drugs associate with apoptosis induction. The combined effects of venetoclax with artesunate at different ratios on apoptosis induction were determined based on morphological observation after staining with acridine orange (AO)/ethidium bromide (EB). The combination indexes (CIs) of the two drugs to induce apoptosis were calculated by the Median-effect equation using CompuSyn software with <1, demonstrating synergy (Fig. S1). The synergistic apoptosis induction was confirmed in THP-1 and MOLM-13 cells using FACS analysis based on Annexin V/PI assay. Based on the sensitivity of each drug alone, we selected different concentrations for the combination in THP-1 and MOLM-13 cells. Venetoclax at 0.1 μM plus artesunate at 1.6 μM induced about 60% of THP-1 cells undergoing apoptosis. Venetoclax at 0.01 μM plus artesunate at 0.4 μM induced about 70% of MOLM-13 cells undergoing apoptosis (Fig. 2C). The DNA distribution assay after staining with PI in fixed cells revealed that the combination of venetoclax with artesunate mainly increased cell number in SubG1 phase (Fig. 2D).

### Bim and Noxa are required for the synergistic apoptosis induction of venetoclax and artesunate combination

**Fig. 3:**
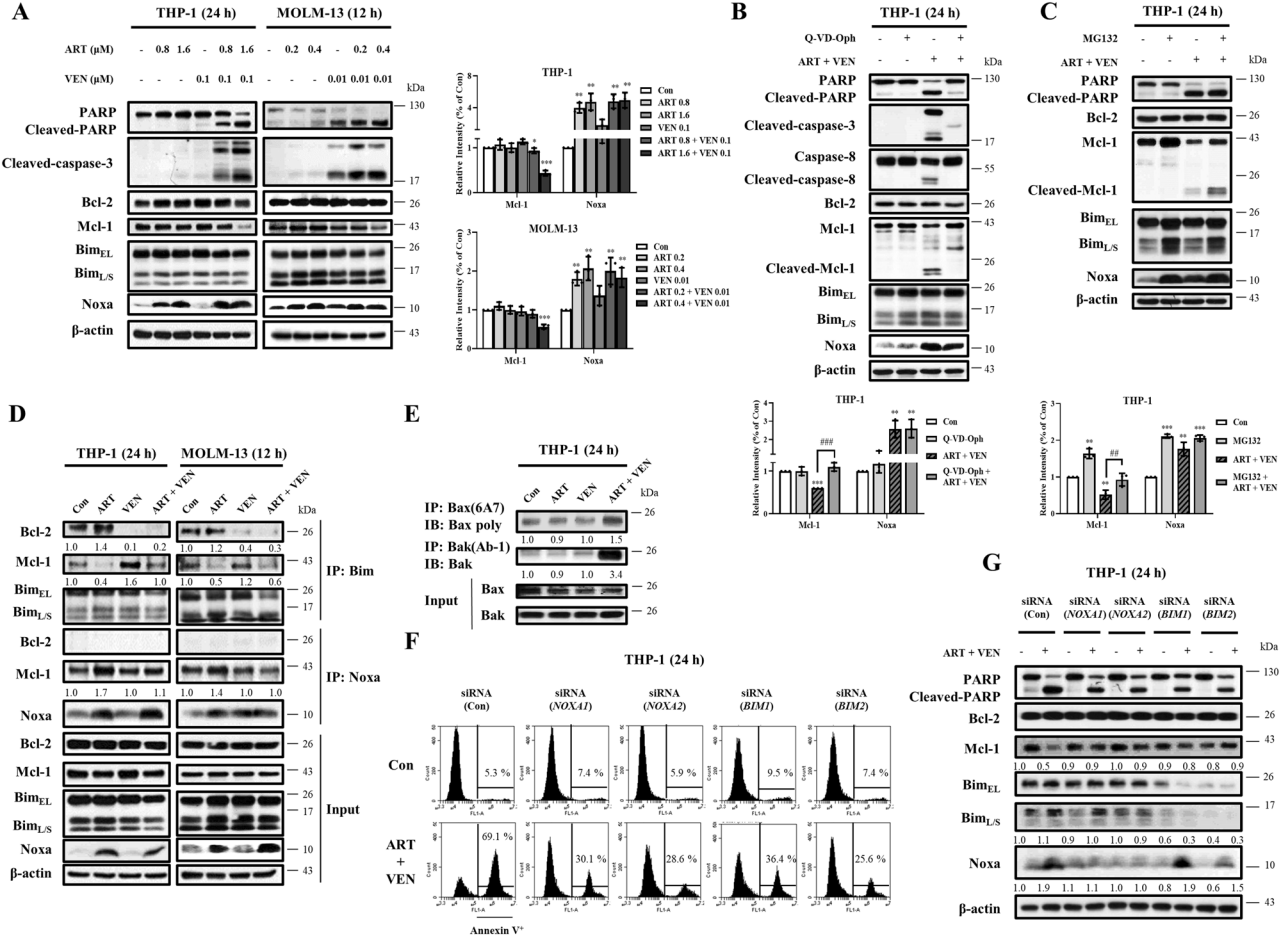
Venetoclax and artesunate induce synergistic apoptosis through the Noxa and Bim-mediated pathway following Mcl-1 reduction. **A** THP-1 and MOLM-13 cells were treated with artesunate and venetoclax at the indicated concentrations and times. Relative levels of the indicated proteins were determined by Western blotting. **B** THP-1 cells were pretreated with 25 μM Q-VD-OPh for 4 h, following by treatment with 1.6 μM artesunate and 0.1 μM venetoclax for 24 h. **C** THP-1 cells were treated with 1.6 μM artesunate and 0.1 μM venetoclax for 18 h, following by treatment with 10 μM MG132 for 6 h. **D** THP-1 cells were treated with 0.8 μM artesunate and 0.1 μM venetoclax for 24 h. MOLM-13 cells were treated with 0.2 μM artesunate and 0.01 μM venetoclax for 12 h. Cell lysates were prepared and immunoprecipitated with anti-Bim or anti-Noxa antibody, and then probed for Bcl-2 and Mcl-1. **E** THP-1 cells treated with 1.6 μM artesunate, 0.1 μM venetoclax and in combination for 24 h. The activated Bak and Bax determined with the anti-Bak(Ab-1) or anti-Bax (6A7) antibody(detecting the active forms). **F** THP-1 cells were transfected with two pairs of *NOXA* and *BIM* siRNAs for 24 h, then treated with 1.6 μM artesunate and 0.1 μM venetoclax for 24 h. Apoptotic cells were quantified using FACS after staining with Annexin V-FITC. **G** Levels of PARP, Bcl-2, Mcl-1, Bim, and Noxa were measured by Western blotting. The column graphs are the mean ± SD of three independent experiments. *P < 0.05; **P < 0.01; ***P < 0.001 compared to the control group by *t*-test. ^##^*P* < 0.01; ^###^*P* < 0.001 by two-way ANOVA test.

The protein levels of Bcl-2, Mcl-1, Bim, and Noxa in THP-1 and MOLM-13 cells treated with venetoclax, artesunate alone, and the combination were measured (Fig. 3A). Artesunate increased the levels of Noxa. The combination kept the induced levels of Noxa with reduced Mcl-1 protein levels in both cell lines (Fig. 3A). To test if the Mcl-1 reduction is due to caspase cleavage or proteasome degradation, we measured the cleaved Mcl-1 fragments and prevention of Mcl-1 reduction in presence of the general caspase inhibitor Q-VD-OPh and the proteasome inhibitor MG132. The cleaved fragment of Mcl-1 was detected in the combination treatment of THP-1 cells and the pretreatment with Q-VD-OPh prevented PARP and Mcl-1 cleavage, but not Noxa induction (Fig. 3B). MG132 alone is toxic and induces apoptosis in a longer time treatment. To separate its apoptosis induction and proteasome inhibition on Mcl-1 regulation, we pretreated THP-1 cells by venetoclax/artesunate for 18 h and then with MG132 for 6 h. MG132 increased the protein levels of parental Mcl-1 and cleaved Mcl-1 in the combination-treated cells, but did not prevent Mcl-1 cleavage (Fig. 3C). We then tested the binding of Bim and Noxa to Bcl-2 and Mcl-1 in THP-1 and MOLM-13 cells treated with artesunate, venetoclax, and the combination. Consistent with reported data [30, 31], venetoclax dissociated Bim from Bcl-2 accompanied with increased Bim binding to Mcl-1. Artesunate increased Noxa binding to Mcl-1 and decreased Bim binding to Mcl-1. The combination released Bim from both Bcl-2 and Mcl-1 proteins (Fig. 3D). Immunoprecipitation (IP) assay revealed that both Bax and Bak were activated by the combination treatment (Fig. 3E). Silencing of *NOXA* and *BIM* using siRNA attenuated the apoptosis induction (Fig. 3F) and Mcl-1 reduction of the combination treatment (Fig. 3G). These data suggest that artesunate/venetoclax combination induce both Bim and Noxa-dependent apoptosis following with Mcl-1 reduction.

### Artesunate and venetoclax enhance cytarabine-induced cytotoxicity through apoptosis-mediated Mcl-1 reduction

We tested the combined effects of cytarabine with venetoclax or artesunate in both THP-1 and MOLM-13 cell lines. The concentrations of cytarabine, venetoclax and artesunate alone were selected with less than 50% of cell growth inhibition for calculating CI. Venetoclax plus cytarabine-induced synergistic growth inhibition in both cell lines with CI < 1 (Fig. S2A). Artesunate did not exhibit obvious synergistic growth inhibition with cytarabine in both cell lines (Fig. S2B). We then compared the apoptosis induction and cell cycle arrest of cytarabine in combination with venetoclax. In THP-1 cells, venetoclax plus cytarabine did not show enhanced apoptosis induction even the concentration of cytarabine increased to 8 μM (Fig. S3A). The S phase arrest was maintained (Fig. S3B). Western blot analysis revealed that Mcl-1 was kept at high levels without PARP cleavage (Fig. S3C). In MOLM-13 cells, cytarabine alone arrested cells in S phase without inducing apoptosis. Venetoclax induced apoptosis alone and the addition of cytarabine had enhanced apoptosis and SubG1 accumulation (Fig. S3B). PARP cleavage and the γ-H2A.X induction by venetoclax were enhanced by addition of cytarabine, while the levels of p-Chk1 induced by cytarabine were attenuated by venetoclax/cytarabine dual combination in MOLM-13 cells, but not in THP-1 cells (Fig. S3C). These data indicate that venetoclax and cytarabine combination has enhanced apoptotic effects in MOLM-13 cells with lower Mcl-1 protein.

**Fig. 4:**
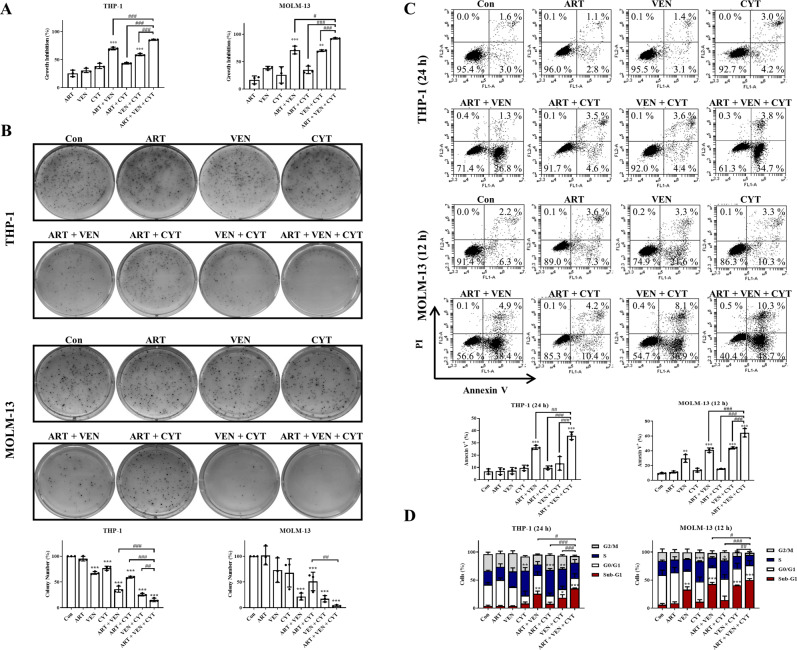
The triple combination of venetoclax, artesunate, and cytarabine is more effective at inhibiting leukemia cell growth and inducing DNA damage. THP-1 cells were treated with 0.8 μM artesunate, 0.1 μM venetoclax, and 0.5 μM cytarabine alone or in combination. MOLM-13 cells were treated with 0.2 μM artesunate, 0.01 μM venetoclax, and 0.005 μM cytarabine alone or in combination. **A** Growth inhibition of the different combination were measured after 72 h treatment. **B** The number of colonies were counted and calculated as a percentage of the control after 14 days. **C** THP-1 cells were treated with 0.8 μM artesunate, 0.1 μM venetoclax, and 8 μM cytarabine alone or in combinations for 24 h. MOLM-13 cells were treated with 0.2 μM artesunate, 0.01 μM venetoclax, and 0.08 μM cytarabine alone or in combination for 12 h. Apoptosis induction measured based on Annexin V/PI staining. **D** Cell cycle assessed after PI staining by flow cytometry. Values are mean ± SD of three independent experiments. *P < 0.05; **P < 0.01; ***P < 0.001 compared to the control group by *t*-test. ^#^*P* < 0.05; ^##^*P* < 0.01; ^###^*P* < 0.001 by two-way ANOVA test.

We compared the effects of triple combination of artesunate, venetoclax, and cytarabine with dual combinations in THP-1 and MOLM-13 cells to inhibit cell growth. We selected the concentrations of each drug alone with less 50% cell growth inhibition for the triple combination. THP-1 cells were treated with 0.8 μM artesunate, 0.1 μM venetoclax, and 0.5 μM cytarabine alone or in the dual and triple combinations. MOLM-13 cells were treated with 0.2 μM artesunate, 0.01 μM venetoclax, and 0.005 μM cytarabine alone or in the dual and triple combinations. Venetoclax plus artesunate or venetoclax plus cytarabine had increased cell growth inhibition abilities comparing to venetoclax or cytarabine alone. The triple combination was the most potent to inhibit cell growth in suspension (72 h treatment, Fig. 4A) and in soft agar growth (14 days treatment, Fig. 4B). To study apoptosis and DNA fragmentation induction in the short time treatment, we increased cytarabine to 8 and 0.08 μM in THP-1 and MOML-13 cells, respectively. Annexin V/PI staining assay indicated that the triple combination had enhanced apoptosis in both cell lines comparing to any dual combination (Fig. 4C). DNA distribution analysis revealed that the triple combination had increased cell number in SubG1 phase comparing to any dual combination (Fig. 4D).

**Fig. 5:**
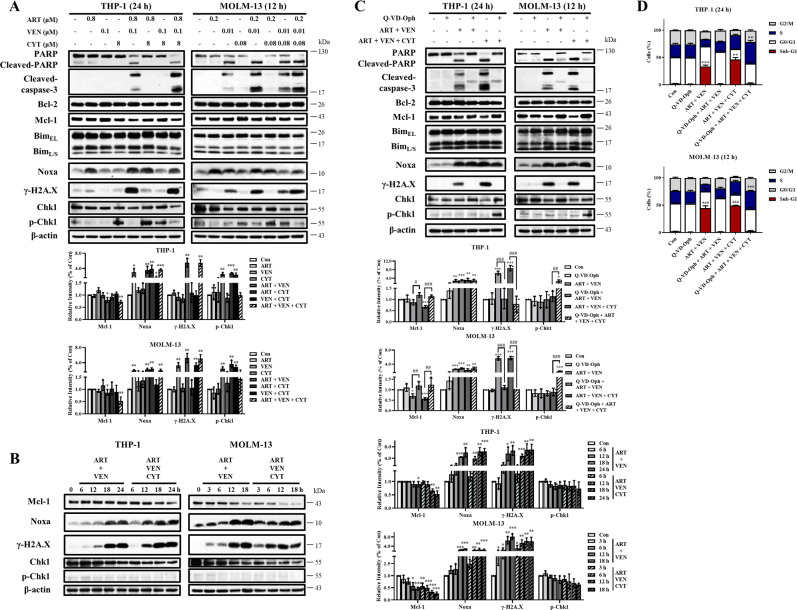
The triple combination has increased DNA damage marker γ-H2A.X with reduced Mcl-1 and repressed p-Chk1. **A** THP-1 cells and MOLM-13 cells were treated as indicated and the protein levels were determined by Western blotting. **B** THP-1 cells were treated with 0.8 μM artesunate, 0.1 μM venetoclax, and 8 μM cytarabine at dual and triple combinations and MOLM-13 cells were treated with 0.2 μM artesunate, 0.01 μM venetoclax, and 0.08 μM cytarabine dual and triple combinations at the indicated times. Western blotting was performed to test protein changes. **C** THP-1 and MOLM-13 cells were pretreated with 25 μM Q-VD-OPh for 4 h, following by the dual and triple combinations. Western blotting was performed to test protein changes. **D** The DNA distribution assessed by flow cytometry after PI staining. The column graphs are the mean ± SD of three independent experiments. *P < 0.05; **P < 0.01; ***P < 0.001 compared to the control group by *t*-test. ^#^*P* < 0.05; ^##^*P* < 0.01; ^###^*P* < 0.001 by two-way ANOVA test.

**Fig. 6:**
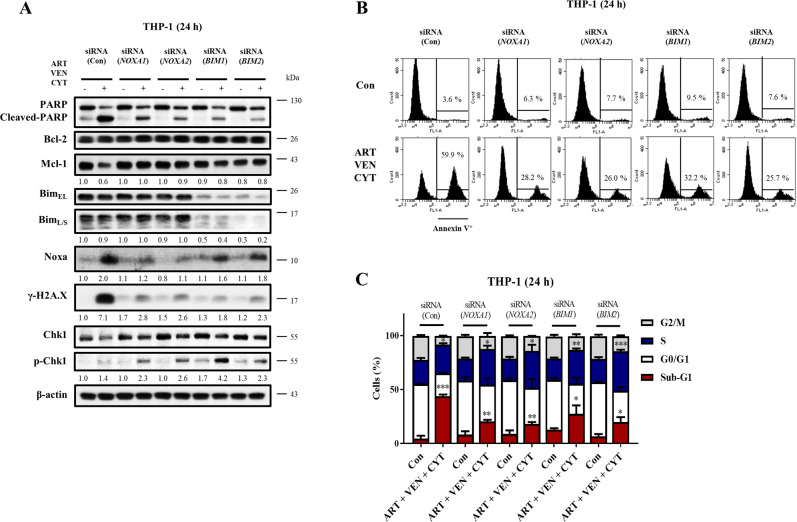
Bim and Noxa-mediated apoptosis contributes to Mcl-1 reduction and p-Chk1 repression in the triple combination. THP-1 cells were transfected with two pairs of *NOXA* and *BIM* siRNAs for 24 h, then treated with the combination of 0.8 μM artesunate, 0.1 μM venetoclax, and 8 μM cytarabine for 24 h. **A** Relative levels of the indicated proteins were determined by Western blotting. **B** Apoptotic cells were quantified using FACS after staining with Annexin V-FITC. **C** Cell cycle distribution was assessed after PI staining by flow cytometry. Values are mean ± SD of three independent experiments. *P < 0.05; **P < 0.01; ***P < 0.001 compared to the control group by *t*-test.

Artesunate-based combinations had induced levels of Noxa. Venetoclax plus artesunate induced PARP and caspase-3 cleavage with increased levels of γ-H2A.X, which were slightly further increased in the triple combination. Cytarabine-based combinations had increased levels of p-Chk1 except in the triple combination. The PARP cleavage associated with the increased levels of γ-H2A.X and the reduced levels of Mcl-1. The levels of Mcl-1 and p-Chk1 in the triple combination were lower than those in cells treated by cytarabine alone or cytarabine plus venetoclax (Fig. 5A). We also noted that the levels of Chk1 protein were decreased in both cell lines associated with the cleaved caspases and PARP. Time-dependent changes of Noxa, Mcl-1, γ-H2A.X, p-Chk1, and Chk1 proteins were measured in both cell lines treated with venetoclax plus artesunate combination and the triple combination. The levels of γ-H2A.X and Noxa were induced fast while the levels of Mcl-1 and Chk1 decreased fast in the triple combination-treated cells than in the dual combination-treated cells (Fig. 5B). The levels of p-Chk1 were not induced in the dual and the triple combination-treated cells (Fig. 5B). The caspase inhibitor Q-VD-OPh blocked Mcl-1 reduction, Chk1 reduction and γ-H2A.X induction in the dual and triple combinations. Importantly, Q-VD-OPh recovered the p-Chk1 induction of cytarabine-based triple combination, but not in the venetoclax/artesunate dual combination (Fig. 5C). Q-VD-OPh blocked SubG1 phase cell accumulation, but increased S phase cell arrest in the triple combination (Fig. 5D). The roles of Noxa and Bim in the triple combination were investigated using siRNA. Silencing either *BIM* or *NOXA* attenuated PARP cleavage and γ-H2A.X induction, blocked Mcl-1 and Chk1 reduction, and recovered p-Chk1 induction (Fig. 6A). Silencing either *BIM* or *NOXA* attenuated the apoptosis induction based on the Annexin V assay (Fig. 6B) and SubG1 cell induction, but not S phase arrest (Fig. 6C). These data suggest that Noxa and Bim-mediated apoptosis enhances cytarabine-induced S phase related DNA damage by blocking Chk1 phosphorylation.

### The triple combination prolongs longer survival of xenografts than dual combinations

**Fig. 7:**
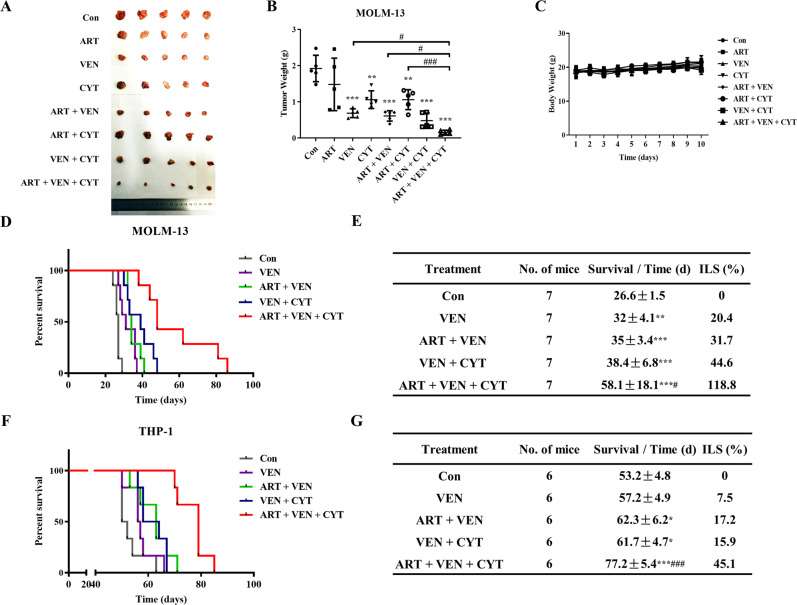
The triple combination significantly improves survival of AML xenografts. **A** Mice inoculated with MOLM-13 cells subcutaneously were treated daily with artesunate (100 mg /kg), venetoclax (100 mg/kg), and cytarabine (50 mg/kg) alone or in combination for 10 days. Tumors were dissected and imaged. **B** Tumors were weighed and calculated as a percentage of the control. **P < 0.01; ***P < 0.001 compared to the control group by *t*-test. ^#^*P* < 0.05; ^###^*P* < 0.001 by two-way ANOVA test. **C** Mice weights during the treatment. **D** Kaplan–Meier survival curve of mice inoculated with MOLM-13 cells through the tail-vein. **E** Average survival time and increase of life span (ILS) of MOLM-13 xenografts. **F** Kaplan–Meier survival curve of mice inoculated with THP-1 cells through the tail-vein. **G** Average survival time and ILS of THP-1 xenografts. *P < 0.05; **P < 0.01; ***, *P* < 0.001 compared to the control group by log-rank test. ^#^*P* < 0.05; ^###^*P* < 0.001 compared to the venetoclax/cytarabine combination by log-rank test.

We first tested the inhibitory effects of the three drugs in different combinations in NOD/SCID mice inoculated with MOLM-13 cells subcutaneously due to its rapid growth. The mice were treated with artesunate (100 mg/kg), venetoclax (100 mg/kg), and cytarabine (50 mg/kg) daily alone or in different combinations for 10 consecutive days. Venetoclax and cytarabine alone were effective to inhibit tumor growth (Fig. 7A). Based on the tumor weights, the triple combination is more effective than venetoclax alone, artesunate/venetoclax and artesunate/cytarabine dual combination but defecting significant difference comparing to the venetoclax/cytarabine dual combination (Fig. 7B). The dual and triple combinations do not influence body weights of mice (Fig. 7C).

We then tested the antileukemia effects in mice with MOLM-13 cells inoculated through tail vein. Since venetoclax alone is effective in the above model, we focused on the venetoclax based therapies for comparison. The mice were treated with venetoclax (100 mg/kg) alone or in combination with artesunate (100 mg/kg) for five days/week for four weeks, or with cytarabine (50 mg/kg) for seven consecutive days in the first week. The mice survived for an average of 27 days without treatment, 32 days with treatment of venetoclax alone, 35 days with treatment of artesunate plus venetoclax, and 38 days with treatment of venetoclax plus cytarabine. An average of 58 days was reached with the triple combination with an increased life span (ILS) of 118.8% (Fig. 7D, E). In the THP-1 cell xenografted mice model, venetoclax alone did not have significant effect, and in combination with cytarabine or artesunate slightly increased the life span by 15.9% and 17.2%. The triple combination prolonged the survival of mice with an ILS% of 45.1% (Fig. 7F, G). These data indicate that the triple combination improves the therapeutic effects of cytarabine plus venetoclax to prolong longer survival of xenografts.

## Discussion

Cytarabine arrests cells in S phase following DNA damage with increased activation of DNA replication kinase p-Chk1. We found that THP-1 cells with high levels of Mcl-1 are less sensitive to cytarabine treatment with fast induction of p-Chk1 (Fig. 1D, E). Silencing of Mcl-1 attenuates p-Chk1 induction with enhanced DNA damage marker γ-H2A.X (Fig. 1F). These data support that Mcl-1 accelerates Chk1 phosphorylation and protect DNA damage.

Mcl-1 is an important antiapoptotic protein and plays a more important role than Bcl-2 in leukemia development and survival maintenance [32]. Mcl-1 binds Bim and Bak to block apoptosis while Bcl-2 binds Bim and Bax to block apoptosis [33]. The free Bim could directly activate Bak/Bax to induce apoptosis [34]. Venetoclax releases Bim from Bcl-2, but with increased Bim binding to Mcl-1 (Fig. 3D). Comparing to MOLM-13 cells, THP-1 cells with higher levels of Mcl-1 are insensitive to venetoclax. These data support that Mcl-1 also plays a negative role in the venetoclax treatment.

Mcl-1 is being considered as a therapeutic target for AML therapy [33]. Several Mcl-1 inhibitors have been developed and assessed in clinical trials, but with observed cardiac side effects [35, 36]. We believe that selectively reduction of Mcl-1 protein in AML cells should be a safe approach to improve venetoclax/cytarabine combination therapy. Mcl-1 usually binds with Bim or Noxa. The Bim binding stabilizes Mcl-1, while Noxa replaces Bim from Mcl-1 and recruits an E3 ligase to degrade Mcl-1 [20, 21]. Upregulated Noxa to release Bim from Mcl-1 has been reported to reduce Mcl-1 protein in the spontaneous apoptotic program of neutrophils [37]. Neutrophils do not express Bcl-2 [38]. Neutrophils with re-expression of Bcl-2 become resistant to apoptosis and have a prolonged life in a similar way to AML cells [39]. Therefore, in AML cells with Bcl-2 expression, the apoptosis induction needs to inhibit Bcl-2 and to induce Noxa for removing Mcl-1 protein. We found that artesunate induces Noxa and in combination with venetoclax synergistically induce apoptosis in AML cells with Mcl-1 reduction.

Cytarabine arrests AML cells in S phase with increased SubG1 and DNA damage marker γ-H2A.X, accompanied by increased p-Chk1 (Fig. 1). We believe that the S phase arrest plays an essential role in cytarabine alone or in cytarabine/venetoclax combination therapies. Venetoclax plus cytarabine exhibit enhanced DNA damage and apoptosis in MOLM-13 cells which have lower Mcl-1 protein (Fig. 1D, S3). The triple combination has increased SubG1 cell induction and cell growth inhibition comparing to dual combinations. This triple combination exhibits dual effects, S phase arrest and enhanced apoptosis (Fig. 4). The caspase inhibitor Q-VD-OPh blocks apoptosis and Mcl-1 reduction, but not S phase arrest, and partly recovers p-Chk1 in the triple combination (Fig. 5). These data support that apoptosis-mediated Mcl-1 reduction and prevention of Chk1 phosphorylation enhance cytarabine cytotoxicity, but not prevent cytarabine S phase arrest. Chk1 is required for normal hematopoiesis and that direct inhibition might cause toxicity to normal cells [40]. A Chk1 inhibitor has been reported to enhance cytarabine cytotoxicity, but it blocks cytarabine-mediated S phase arrest [41]. In this triple combination we provide an indirect way of preventing Chk1 phosphorylation without influencing S phase arrest of cytarabine treatment. Recently it has been reported that Chk1 can be cleaved by caspases in apoptotic cells [42, 43]. We found that the levels of Chk1 were decreased by the combination treatment and the Chk1 reduction was blocked by the caspase inhibitor Q-VD-OPh (Fig. 5C). Therefore, this Noxa/Bim-mediated apoptotic program seems to have dual effects to inactivate Chk1, inducing Chk1 cleavage and preventing Chk1 phosphorylation through reducing Mcl-1 protein. This triplet should be more effective and less toxic than directly adding a Chk1 inhibitor, which needs to be tested and compared in future.

**Fig. 8:**
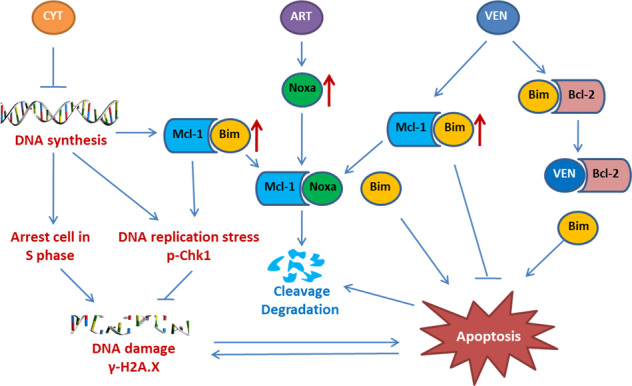
Mechanistic cascade of cytarabine (CYT), venetoclax (VEN), and artesunate (ART) triple combination to induce augmented death of AML cells.

Overall, we provide an innovative triple combination of artesuante, venetoclax and cytarabine to improve antileukemia effects of AML therapy. In this triplet, we found that (1) venetoclax releases Bim from Bcl-2 accompanied with increased Bim binding and stabilization of Mcl-1; (2) artesunate induces Noxa, which replaces Bim from Mcl-1; (3) venetoclax plus artesunate release Bim from both Bcl-2 and Mcl-1 and lead to synergistic apoptosis following Mcl-1 reduction; (4) cytarabine arrests cells in S phase and induces replication stress kinase p-Chk1 to counteract DNA damage; (5) The Mcl-1 reduction induced by venetoclax plus artesunate diminishes Chk1 phosphorylation and enhances DNA damage of cytarabine treatment (Fig. 8). All three drugs are used in clinic, and this triplet could be easily translated into clinical trials to test the efficacy in AML patients.

## Materials and methods

### Reagents

Artesunate, MG132, EB, and AO were purchased from Sigma-Aldrich Inc. Venetoclax (ABT-199) was purchased from Selleck Chemical. Cytarabine and Q-VD-OPh were purchased from MedChemExpress. Antibodies to PARP and caspase-8 were obtained from BD Biosciences. Antibodies to Bcl-2 (C-2), Actin (C-2), Mcl-1 (S-19), Mcl-1 (G-7), Bax (6A7) and Chk1 (G-4) were obtained from Santa Cruz Biotechnology, Inc. Antibodies to Mcl-1 (D35A5), Bim (C34C5), Bak (D4E4), Bax poly, Noxa (D8L7U), Cleaved Caspase-3 (Asp175), Phospho-Chk1 (Ser345) (133D3), and Phospho-Histone H2A.X (Ser139) (20E3) were obtained from Cell Signaling Technology, Inc. Antibodies to Noxa were obtained from Abcam, Inc. Bak (Ab-1) was obtained from Merck Millipore. *NOXA*(sc-37305), *BIM*(sc-29802), *MCL1*(sc-35877)siRNA, and a control siRNA were purchased from Santa Cruz Biotechnology, Inc. *NOXA*(s10708), *BIM*(s195011), *MCL1*(s8583)siRNA was purchased from Thermo Fisher Scientific.

### Cell lines

U937, Mono Mac 6, THP-1, MOLM-13, and HL-60 cell lines were used and obtained as previously reported [44, 45]. These cells were cultured in RPMI 1640 medium supplemented with 100 units/mL penicillin, 100 μg/mL streptomycin, 1 mmol/L L-glutamine, and 10% (v/v) heat-inactivated fetal bovine serum (FBS).

### Detection of apoptosis and cell cycle

Apoptotic cells were determined by morphologic observation after staining with AO/EB and by fluorescence-activated cell sorting (FACS) analysis after staining with Annexin V-PI (BD Biosciences) as previously reported [45]. The cell cycle was analyzed after staining with PI. Cells were fixed in ice-cold 70% ethanol and stored at −20 °C for 24 h. The fixed cells were then washed with PBS at room temperature, treated with 1 mg/mL RNase at 37 °C for 0.5 h. Then cells were stained with PI, and the DNA content was quantified by flow cytometry with an excitation wavelength of 488 nm and emission wavelength of 625 nm. The resulting data was analyzed using CELLQuest software.

### Western blot analysis, activation of Bax and Bak, and siRNA interfering

These experiments were performed as we previously reported [44].

### IP assay

Cells treated with different drugs were lysed with NP-40 lysis buffer [50 mmol/L Tris-HCl (pH 7.5), 150 mmol/L NaCl, 5 mmol/L EDTA, 0.5% NP-40, 50 mmol/L NaF, 0.2 mmol/L Na_3_VO_4_, and 1 mmol/L DTT] for 120 min. Total protein (400 µg) was first precleared with 20 µl protein A/G plus-agarose (Santa Cruz Bio-technology) and then subjected to IP with anti-Bim antibody (Cell Signaling Technology, Inc.) or anti-Noxa antibody (Cell Signaling Technology, Inc.) at 4 °C for overnight. Twenty microliter of protein A/G plus-agarose beads was added and incubated for 2 h to pull down protein-antibody complexes. The beads were spun, washed four times with NP-40 lysis buffer, resuspended in 2X SDS sample buffer [50 mM Tris-HCl (pH 6.8), 2% SDS, 10% glycerol, 5% β-mercaptoethanol], and heated at 98 °C for 5 min for analysis by SDS-polyacrylamide gel electrophoresis and Western blotting.

### Colony forming assay

Two layers of soft agar were used to test the colony formation. The drugs were mixed in the bottom layer of agar (0.5%), and 5000 cells were mixed in the upper layer of agar (0.3%). Dishes were incubated at 37 °C in humidified atmosphere containing 5% CO_2_/95% air for 14–16 days. Colony units were visualized using an inverted microscope, and the number of colonies containing >50 cells was counted. Colonies were stained with MTT solution and photographed.

### Xenografts

These experiments were conducted in accordance with the requirements of animal experiment ethics committee of Shenyang Pharmaceutical University. NOD/SCID mice (8 weeks, Beijing Weitonglihua) were injected with 5 × 10^6^ log-phase MOLM-13 cells subcutaneously in the right flank [45]. Mice with tumors of about 100 mm^3^ were randomized to eight groups of five mice each and were treated with artesunate (100 mg/kg, i.p.), venetoclax (100 mg/kg, p.o.), or cytarabine (50 mg/kg, i.p.) alone or in combination for 10 days. Artesunate was dissolved in 10% (5% NaHCO_3_) + 90% saline, venetoclax in 10% ethanol + 30% PEG + 60% Phosal 50 PG, and cytarabine in saline. Control mice received the vehicle (10% ethanol + 30% PEG + 60% Phosal 50 PG) through the same route with the same schedule. The tumor in each mouse was dissected at day 11 and weighed. In the second experiment, 35 NOD/SCID mice (8 weeks) were injected with 2 × 10^6^ log-phase MOLM-13 cells via the lateral tail vein. Three days after leukemic cell injection, the mice were divided randomly into five groups and treated with artesunate (100 mg/kg, i.p.) and venetoclax (100 mg/kg, p.o.) for five days a week for four continuous weeks, and cytarabine (50 mg/kg, i.p.) for seven consecutive days in the first week, alone or in combination. Control mice received the vehicle through the same route with the same schedule. Survival days of each group were monitored and compared with the Kaplan–Meier survival analysis, and the increase of life span (ILS) was calculated as (treated group−control group)/control group × 100%. In the third experiment, 30 NOD/SCID mice (8 weeks) were injected with 2 × 10^6^ log-phase THP-1 cells via the lateral tail vein and treated as described in the second experiment.

### Statistical analysis

Experiments were performed in triplicate, and the data were presented as the mean ± SD. Statistical analyses were conducted using Microsoft Excel (Microsoft Excel, Microsoft Corp) and Prism software v8.0 (GraphPad Software, La Jolla, CA, USA). The student *t*-test was adopted for significance between differences in the means of two groups. The two-way ANOVA test was performed to compare groups in the combination experiments. Overall probability of survival was estimated via the Kaplan–Meier method, and statistical analysis was performed using log-rank testing. A *p* value of <0.05 was considered statistically significant.

## Supplementary information

Supplementary Figure Legends

Supplementary Figures

aj-checklist

Uncut Western blots

## Data Availability

All data needed to support the conclusions are included in this published article and supplementary materials.
