# Supplementary material for: Artesunate improves venetoclax plus cytarabine AML cell targeting by regulating the Noxa/Bim/Mcl-1/p-Chk1 axis
Source: Cell Death Dis. 2022 Apr 20;13(4):379. doi: 10.1038/s41419-022-04810-z (PMC9021233; doi:10.1038/s41419-022-04810-z)
Supplement: Supplementary file 1 — Supplementary Figure Legends [file 41419_2022_4810_MOESM1_ESM.docx]

**Supplementary Figure legends**

**Figure S1. Artesunate and venetoclax induce synergistic apoptosis in AML cells**

**(A)** U937, Mono Mac 6, THP-1, MOLM-13, and HL-60 cells were treated with artesunate and venetoclax in different combinations for 24 h. Apoptotic cells were determined based on morphological observation using a fluorescence microscope after staining with AO and EB. Values shown are mean ± SD of three independent experiments. **(B)** The combination index (CI) calculated by median dose effect analysis using CompuSyn software. CI values of less than 1.0 (horizontal line) correspond to a synergistic interaction.

**Figure S2. Venetoclax, but not artesunate, in combination with cytarabine synergistically inhibit cell growth in AML cells**

**(A)** THP-1 and MOLM-13 cells were treated with cytarabine in combination with venetoclax at the indicated concentrations for 72 h. **(B)** THP-1 and MOLM-13 cells were treated with cytarabine in combination with artesunate at the indicated concentrations for 72 h. Cell growth inhibition rates were determined, and the CIs were analyzed using CompuSyn software.

**Figure S3. Cytarabine in combination with venetoclax induce enhanced DNA damage marker γ-H2A.X without repression of p-Chk1**

THP-1 and MOLM-13 cells were treated with cytarabine in combination with venetoclax at the indicated concentrations for 24 h and 12 h, respectively. **(A)** Apoptotic cells were determined by FACS after staining with Annexin V /PI. **(B)** Cell cycle distribution was assessed by FACS after PI staining. **(C)** Protein levels were determined by Western blotting. Column graphs shown are mean ± SD of three independent experiments.*, *P* < 0.05; **, *P* < 0.01; ***, *P* < 0.001 compared to the control group by t-test. ^#^, *P*< 0.05 by two-way ANOVA test.
