# Supplementary material for: Artesunate improves venetoclax plus cytarabine AML cell targeting by regulating the Noxa/Bim/Mcl-1/p-Chk1 axis
Source: Cell Death Dis. 2022 Apr 20;13(4):379. doi: 10.1038/s41419-022-04810-z (PMC9021233; doi:10.1038/s41419-022-04810-z)
Supplement: Supplementary file 2 — Supplementary Figures [file 41419_2022_4810_MOESM2_ESM.pptx]

## Slide 1
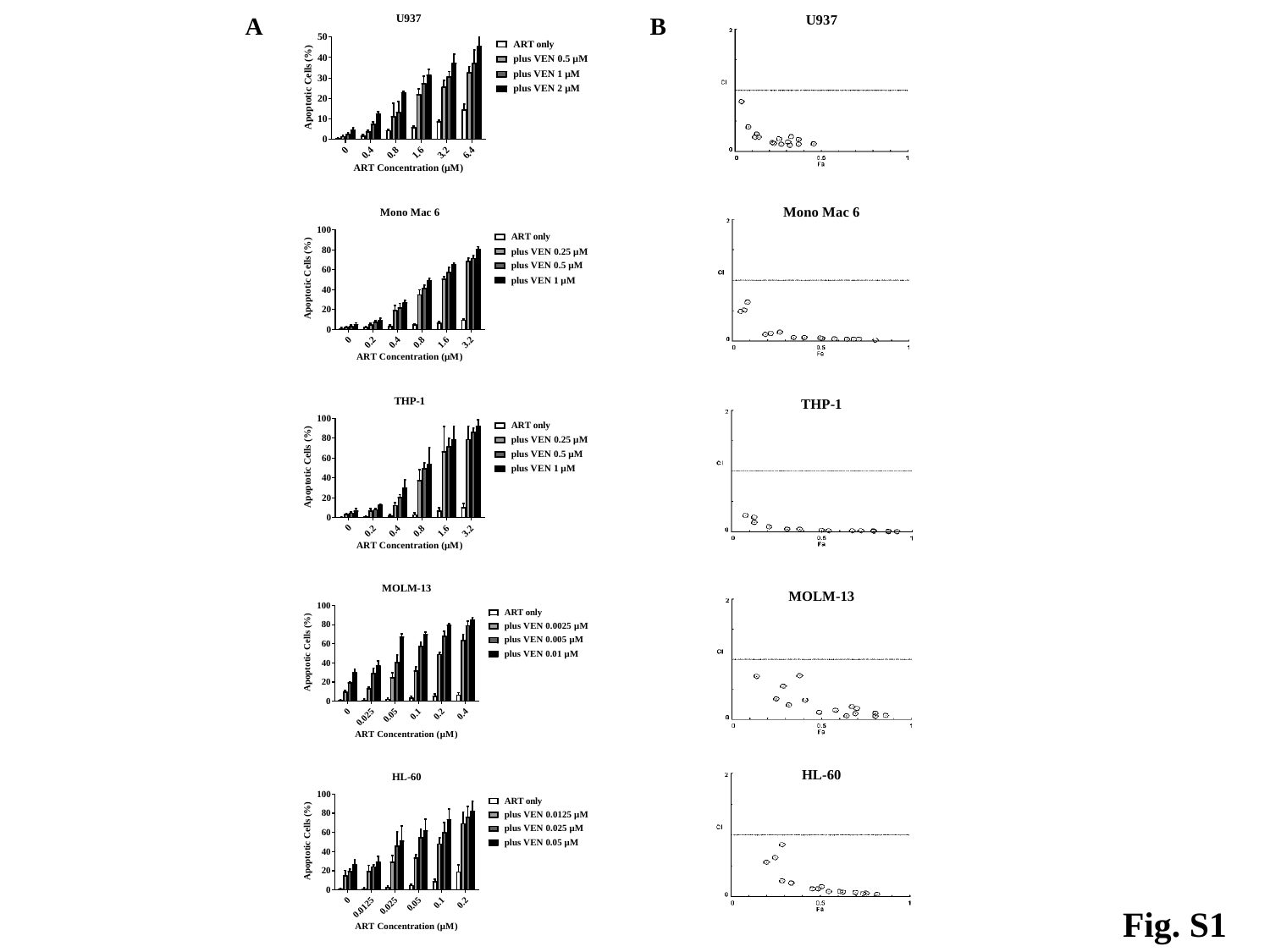

A
B
U937
Mono Mac 6
THP-1
MOLM-13
HL-60
Fig. S1

## Slide 2
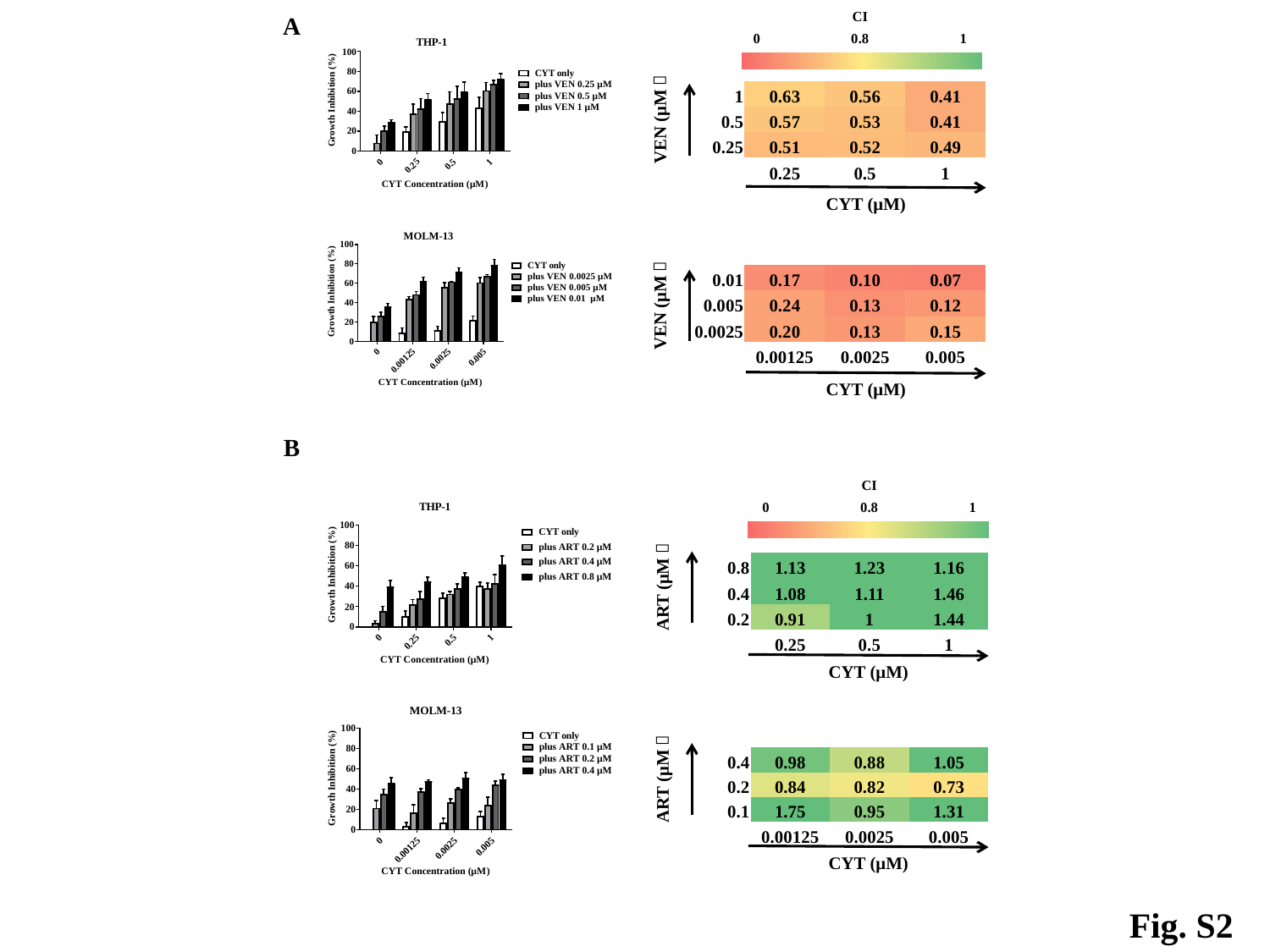

CI
A
0
0.8
1
VEN (μM）
| 1 | 0.63 | 0.56 | 0.41 |
| --- | --- | --- | --- |
| 0.5 | 0.57 | 0.53 | 0.41 |
| 0.25 | 0.51 | 0.52 | 0.49 |
| | 0.25 | 0.5 | 1 |
CYT (μM)
VEN (μM）
| 0.01 | 0.17 | 0.10 | 0.07 |
| --- | --- | --- | --- |
| 0.005 | 0.24 | 0.13 | 0.12 |
| 0.0025 | 0.20 | 0.13 | 0.15 |
| | 0.00125 | 0.0025 | 0.005 |
CYT (μM)
B
CI
0
0.8
1
ART (μM）
| 0.8 | 1.13 | 1.23 | 1.16 |
| --- | --- | --- | --- |
| 0.4 | 1.08 | 1.11 | 1.46 |
| 0.2 | 0.91 | 1 | 1.44 |
| | 0.25 | 0.5 | 1 |
CYT (μM)
ART (μM）
| 0.4 | 0.98 | 0.88 | 1.05 |
| --- | --- | --- | --- |
| 0.2 | 0.84 | 0.82 | 0.73 |
| 0.1 | 1.75 | 0.95 | 1.31 |
| | 0.00125 | 0.0025 | 0.005 |
CYT (μM)
Fig. S2

## Slide 3
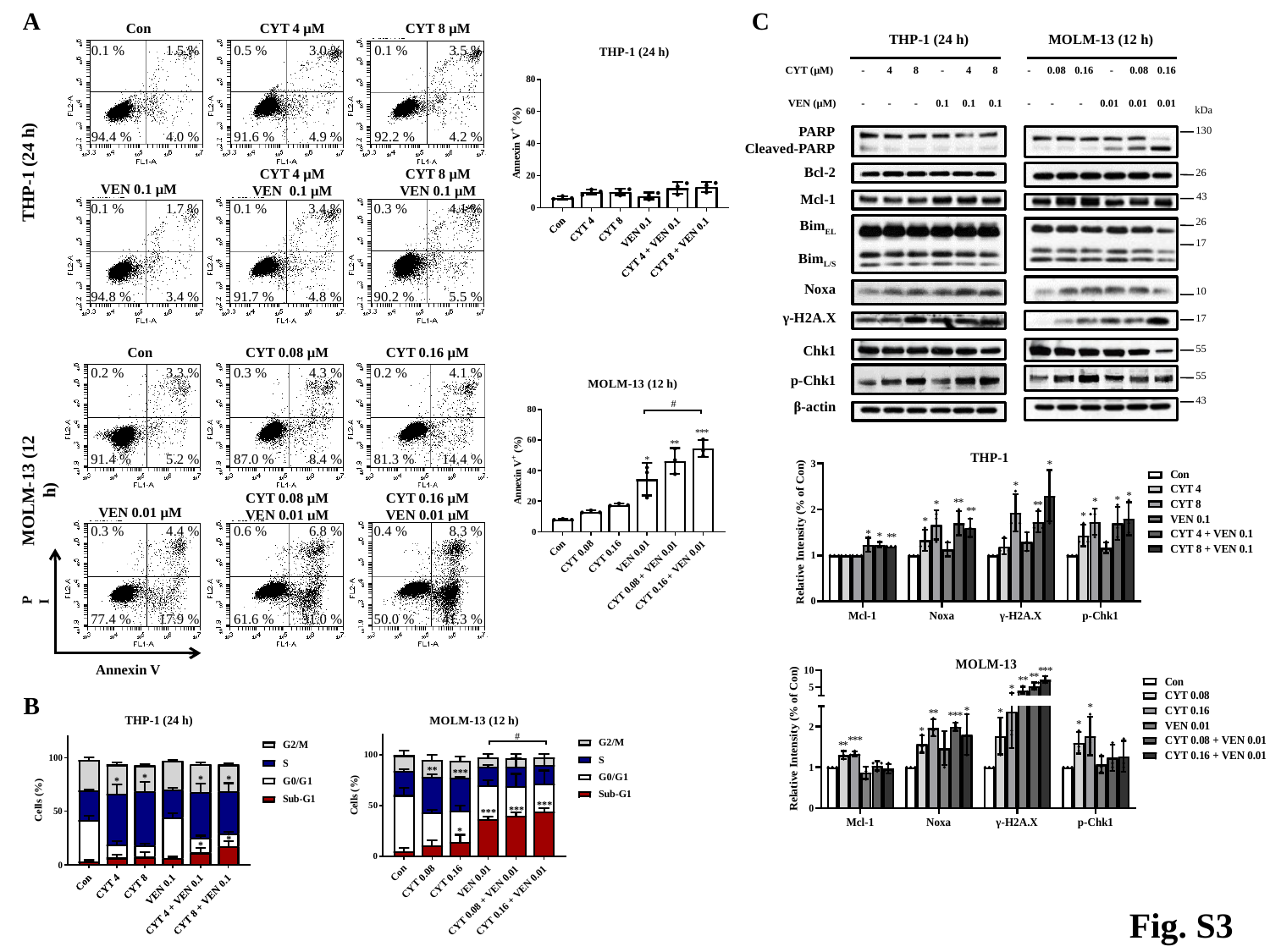

A
C
Con
CYT 4 μM
CYT 8 μM
0.1 %
1.5 %
0.5 %
3.0 %
0.1 %
3.5 %
THP-1 (24 h)
94.4 %
4.0 %
91.6 %
4.9 %
92.2 %
4.2 %
CYT 4 μM
VEN 0.1 μM
CYT 8 μM
VEN 0.1 μM
VEN 0.1 μM
0.1 %
1.7 %
0.1 %
3.4 %
0.3 %
4.1 %
94.8 %
3.4 %
91.7 %
4.8 %
90.2 %
5.5 %
THP-1 (24 h)
MOLM-13 (12 h)
CYT (μM)
-
4
8
-
4
8
-
0.08
0.16
-
0.08
0.16
VEN (μM)
-
-
-
0.1
0.1
0.1
-
-
-
0.01
0.01
0.01
kDa
PARP
Cleaved-PARP
130
Bcl-2
26
43
Mcl-1
26
BimEL
17
BimL/S
Noxa
10
γ-H2A.X
17
55
Chk1
55
p-Chk1
43
β-actin
Con
CYT 0.08 μM
CYT 0.16 μM
0.2 %
3.3 %
0.3 %
4.3 %
0.2 %
4.1 %
MOLM-13 (12 h)
91.4 %
5.2 %
87.0 %
8.4 %
81.3 %
14.4 %
CYT 0.08 μM
VEN 0.01 μM
CYT 0.16 μM
VEN 0.01 μM
VEN 0.01 μM
0.3 %
4.4 %
0.6 %
6.8 %
0.4 %
8.3 %
77.4 %
17.9 %
61.6 %
31.0 %
50.0 %
41.3 %
PI
Annexin V
B
Fig. S3
