# Supplementary material for: Artesunate improves venetoclax plus cytarabine AML cell targeting by regulating the Noxa/Bim/Mcl-1/p-Chk1 axis
Source: Cell Death Dis. 2022 Apr 20;13(4):379. doi: 10.1038/s41419-022-04810-z (PMC9021233; doi:10.1038/s41419-022-04810-z)
Supplement: Supplementary file 4 — Uncut Western blots [file 41419_2022_4810_MOESM4_ESM.pdf]

Fig. 1D

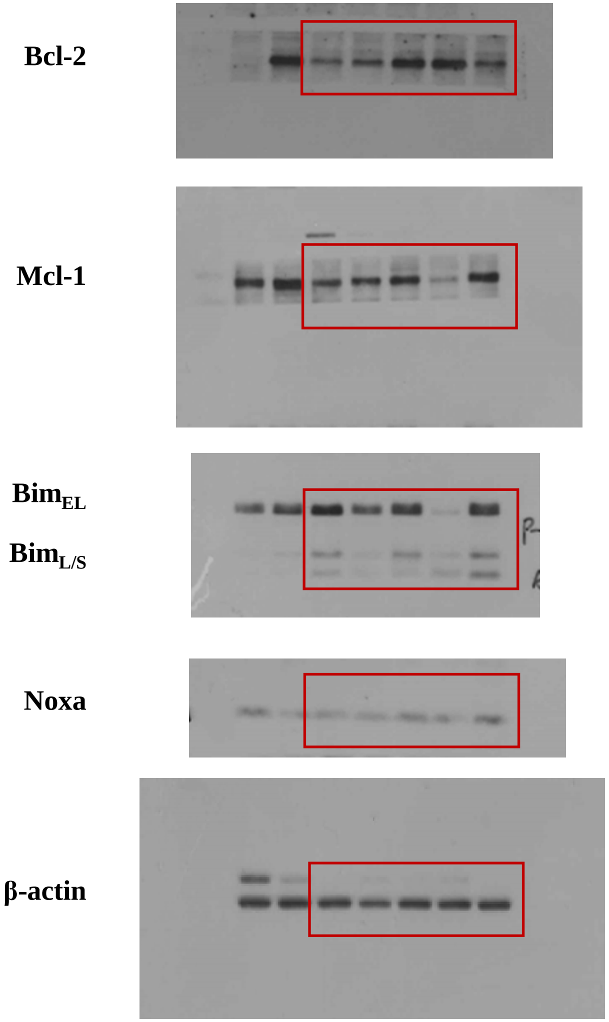

Fig. 1E

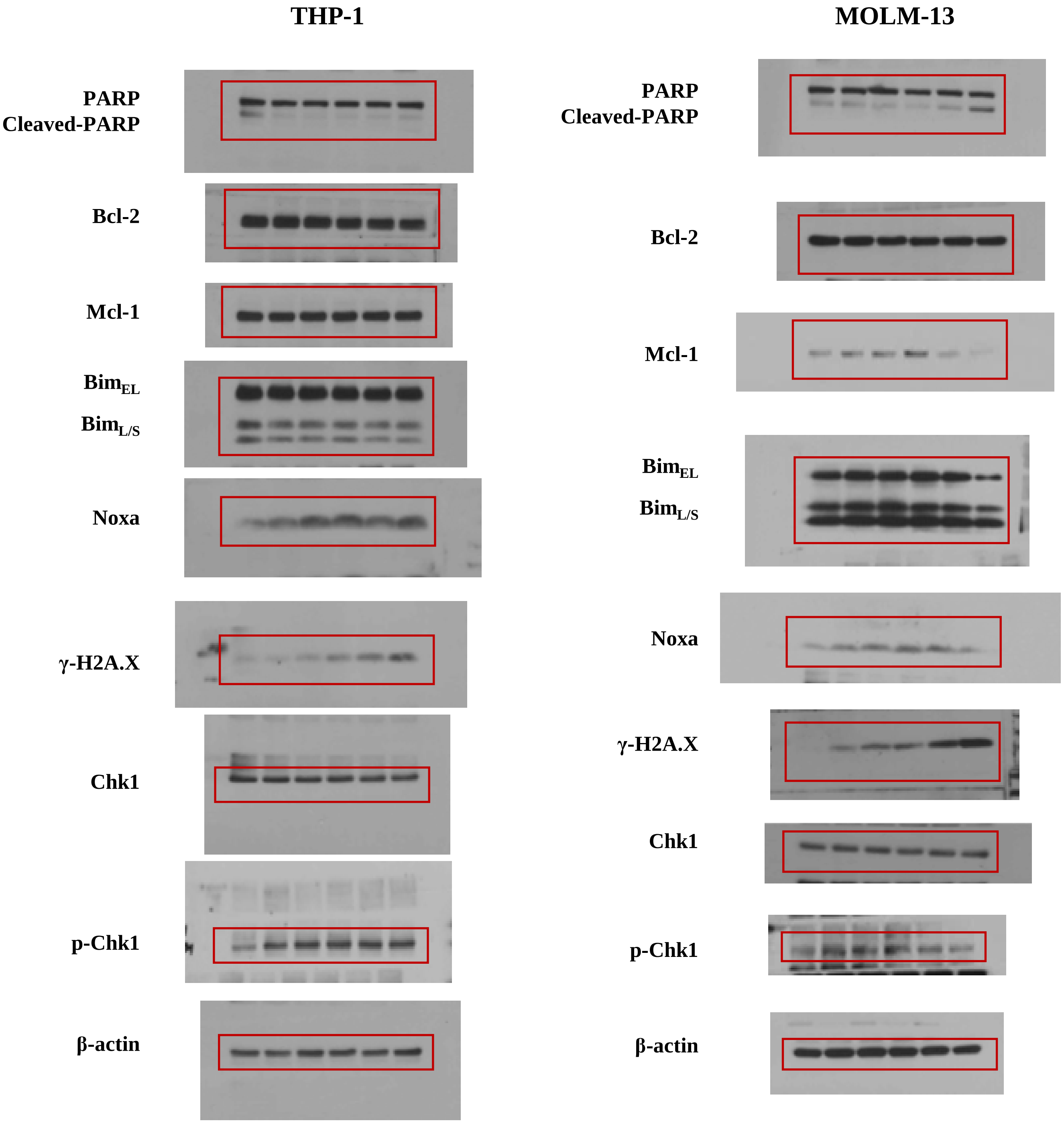

THP-1
MOLM-13
PARP
Cleaved-PARP
Bcl-2
Mcl-1
Bim$_{EL}$
Bim$_{L/S}$
Noxa
γ-H2A.X
Chk1
p-Chk1
β-actin
PARP
Cleaved-PARP
Bcl-2
Mcl-1
Bim$_{EL}$
Bim$_{L/S}$
Noxa
γ-H2A.X
Chk1
p-Chk1
β-actin

Fig. 1F

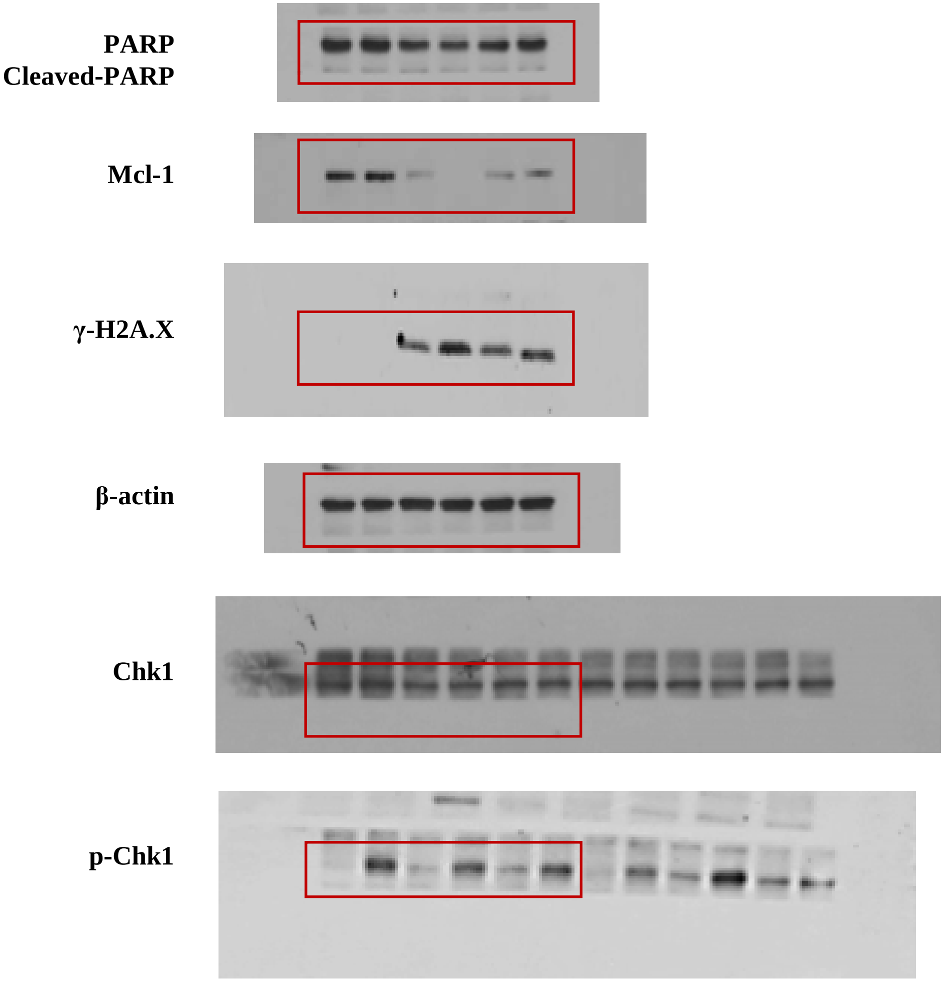

Fig. 3A

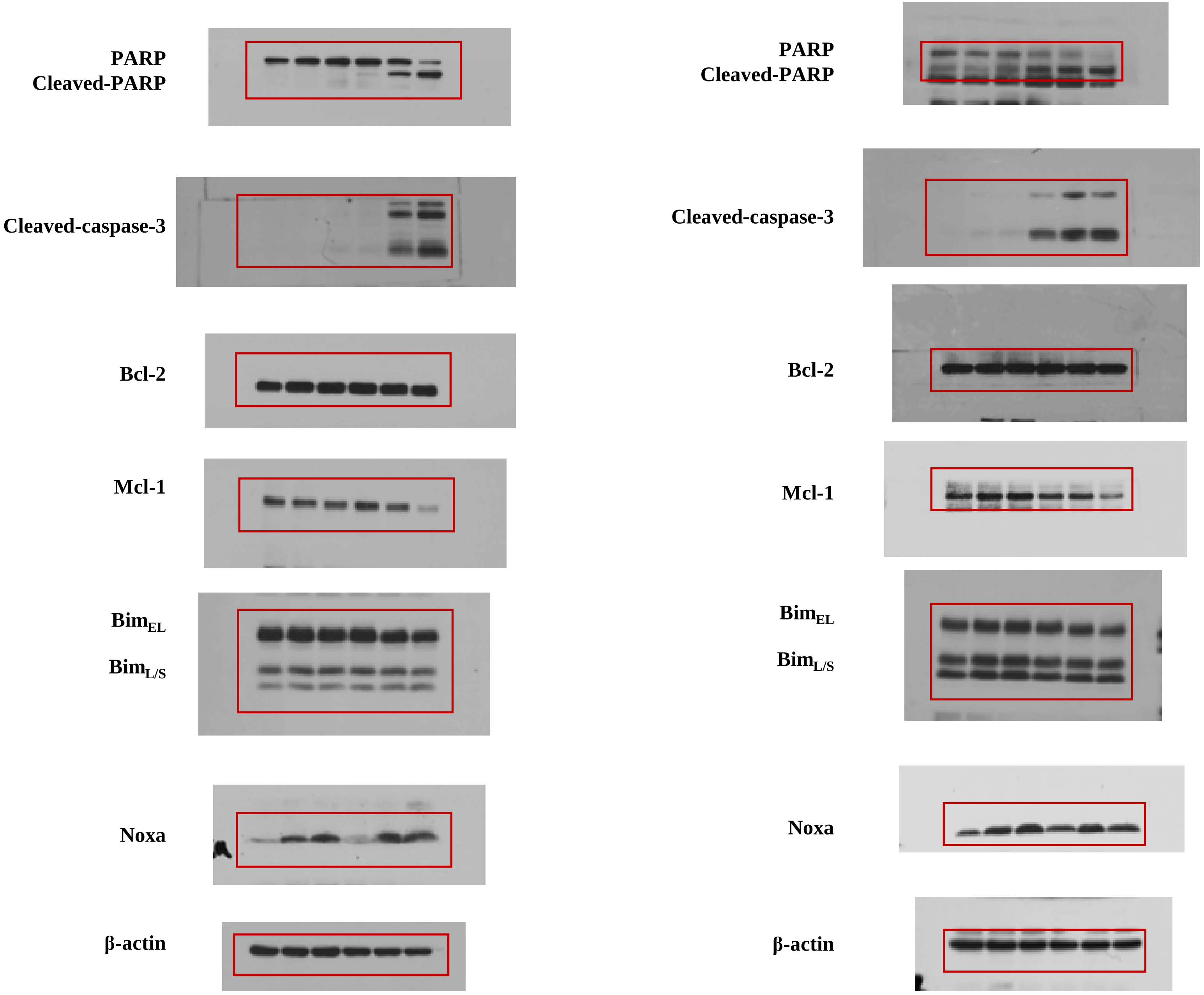
THP-1
MOLM-13
PARP
Cleaved-PARP
Cleaved-caspase-3
Bcl-2
Mcl-1
Bim_{EL}
$Bim_{L/S}$
Noxa
β-actin
PARP
Cleaved-PARP
Cleaved-caspase-3
Bcl-2
Mcl-1
Bim_{EL}
$Bim_{L/S}$
Noxa
β-actin

Fig. 3B

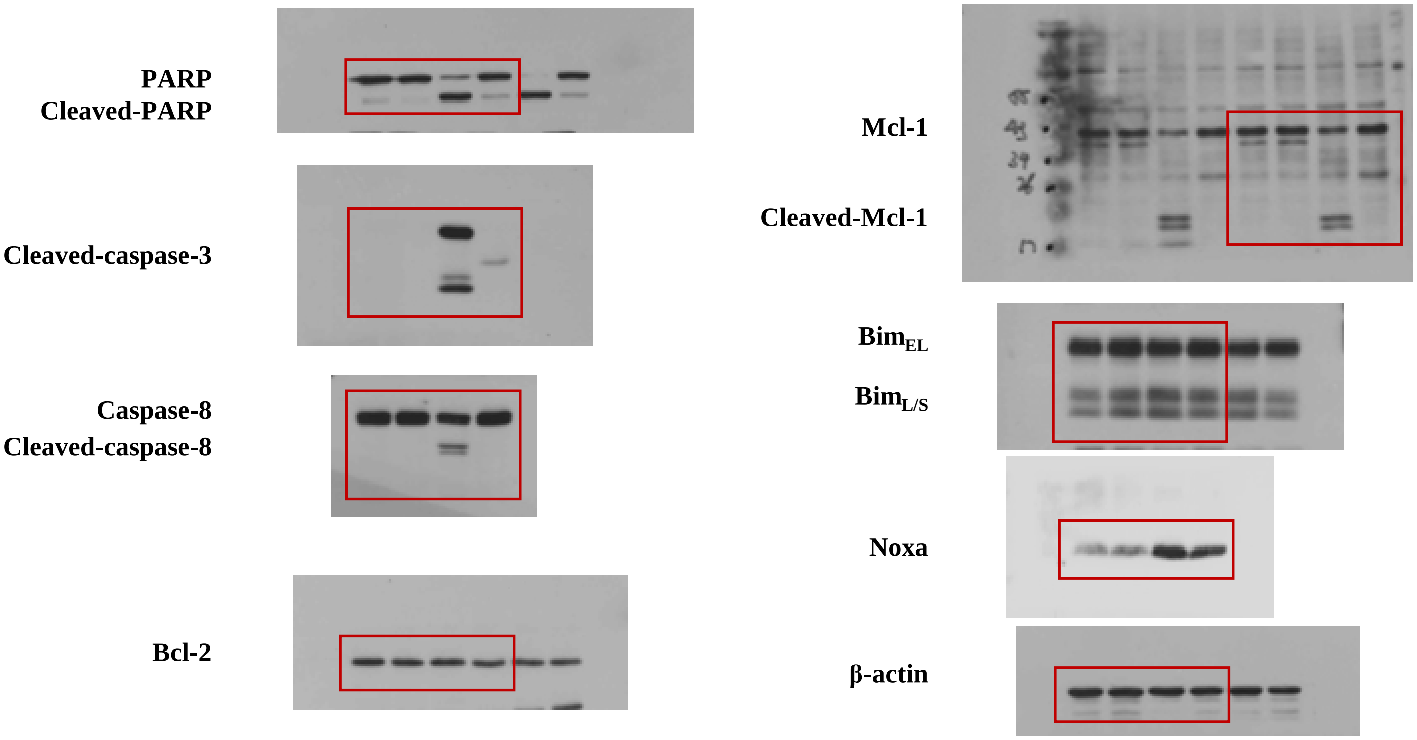
PARP
Cleaved-PARP
Cleaved-caspase-3
Caspase-8
Cleaved-caspase-8
Bcl-2
Mcl-1
Cleaved-Mcl-1
Bim_{EL}
$Bim_{L/S}$
Noxa
β-actin

Fig. 3C

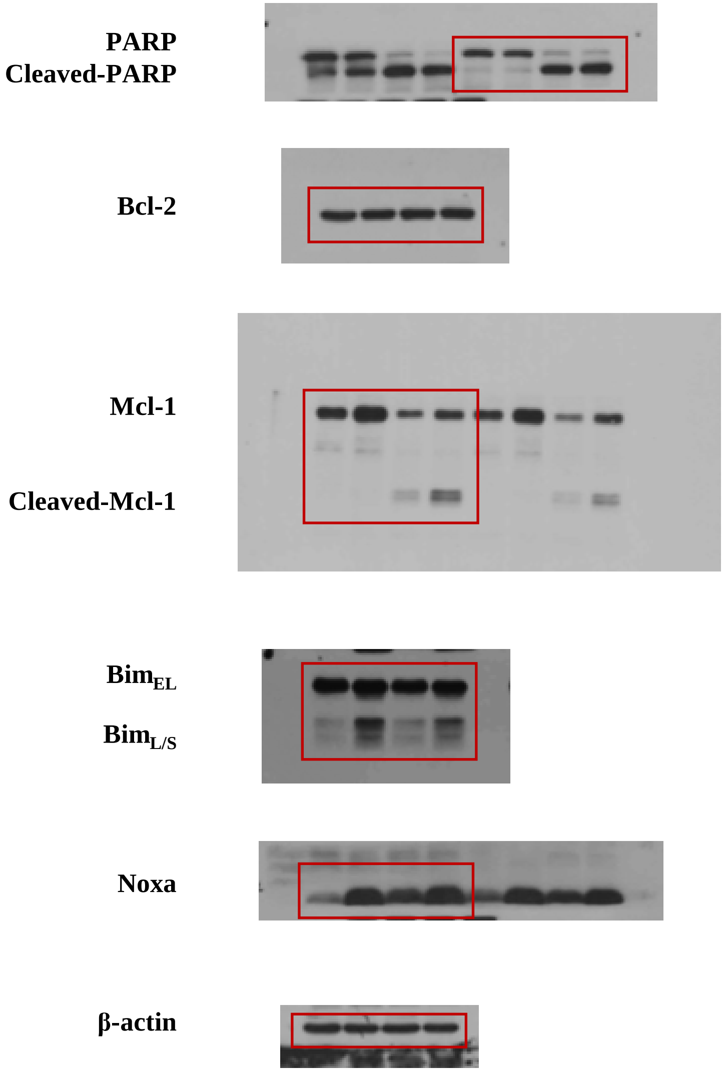

PARP
Cleaved-PARP
Bcl-2
Mcl-1
Cleaved-Mcl-1
Bim$_{EL}$
Bim$_{L/S}$
Noxa
β-actin

Fig. 3D

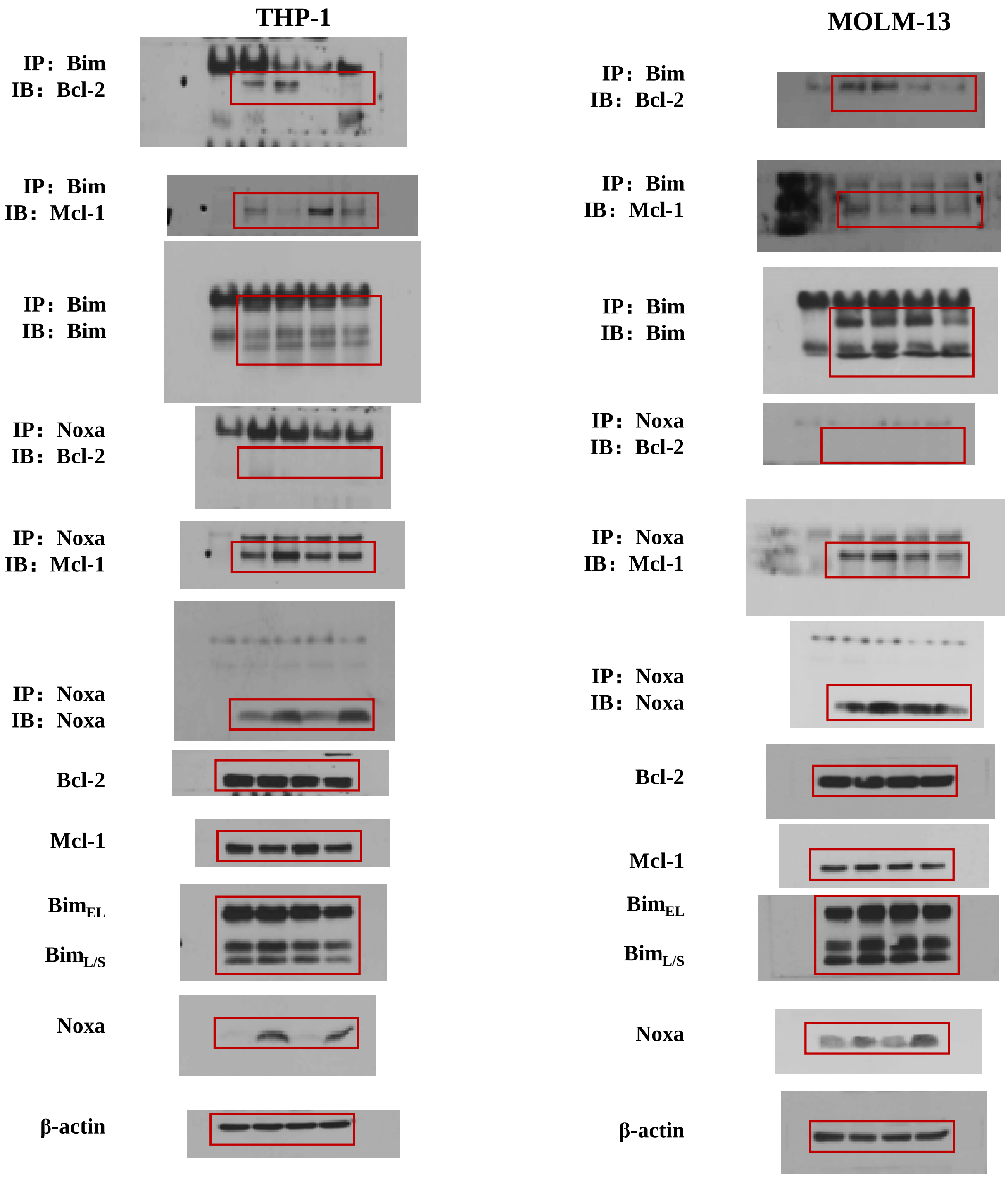
THP-1
MOLM-13
IP: Bim
IB: Bcl-2
IP: Bim
IB: Mcl-1
IP: Bim
IB: Bim
IP: Noxa
IB: Bcl-2
IP: Noxa
IB: Mcl-1
IP: Noxa
IB: Noxa
Bcl-2
Mcl-1
Bim$_{EL}$
Bim$_{L/S}$
Noxa
β-actin

Fig. 3E

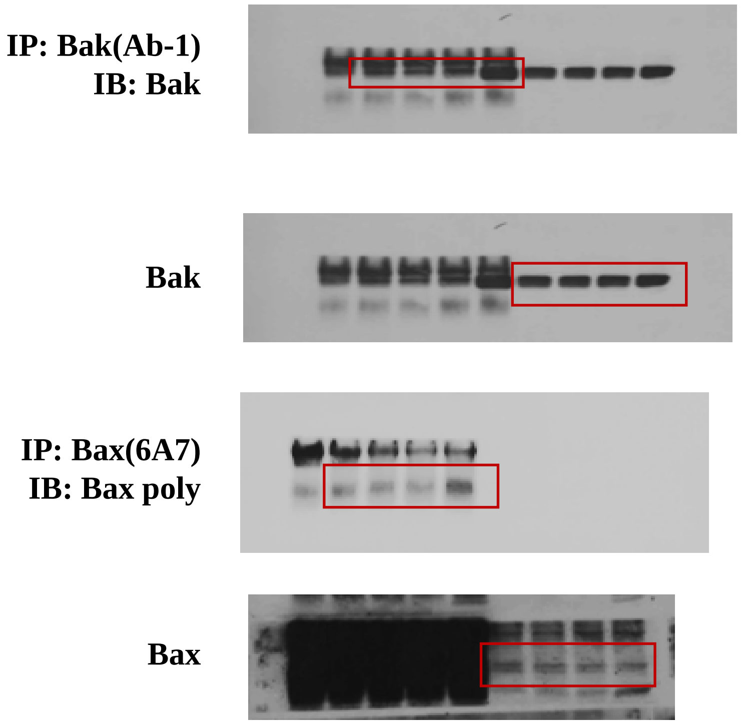

Fig. 3G

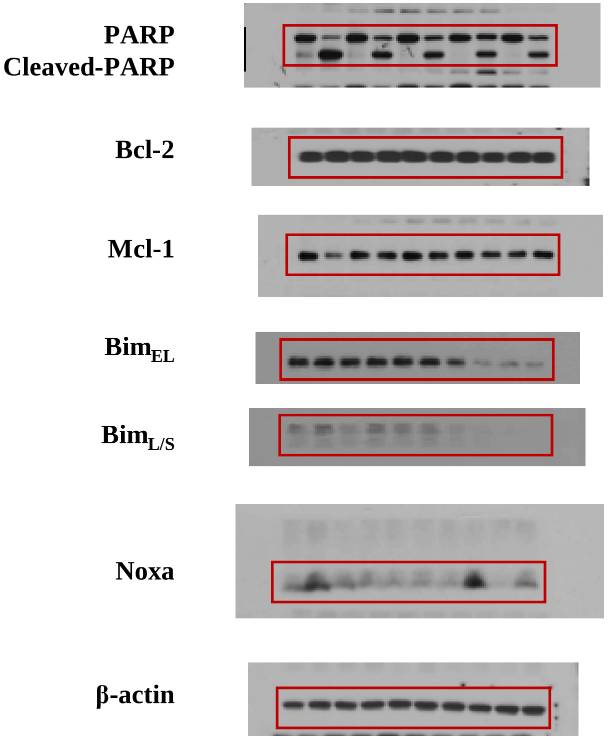

Fig. 5A

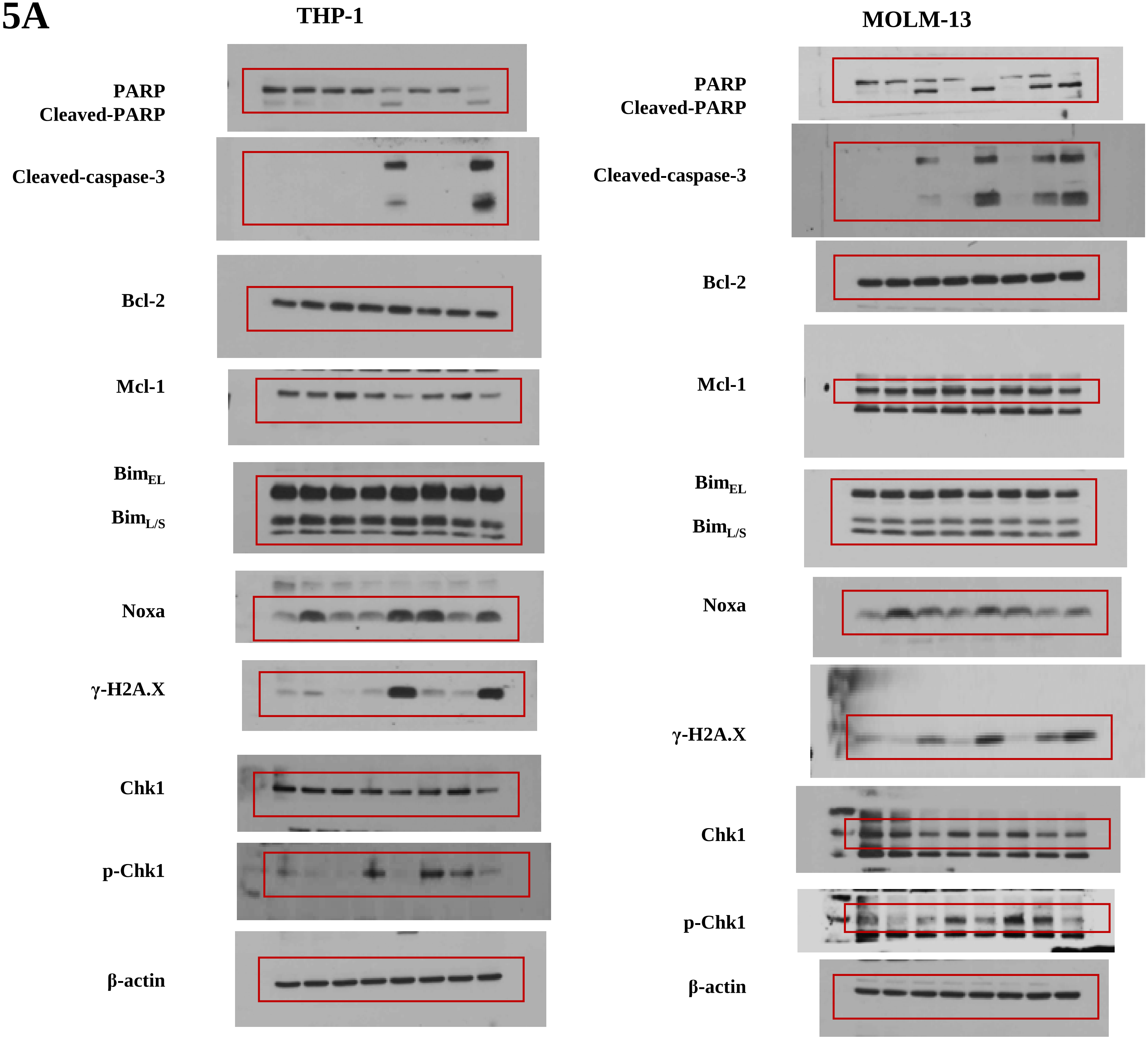
THP-1
MOLM-13
PARP
Cleaved-PARP
Cleaved-caspase-3
Bcl-2
Mcl-1
Bim_{EL}
$Bim_{L/S}$
Noxa
γ-H2A.X
Chk1
p-Chk1
β-actin

Fig. 5B

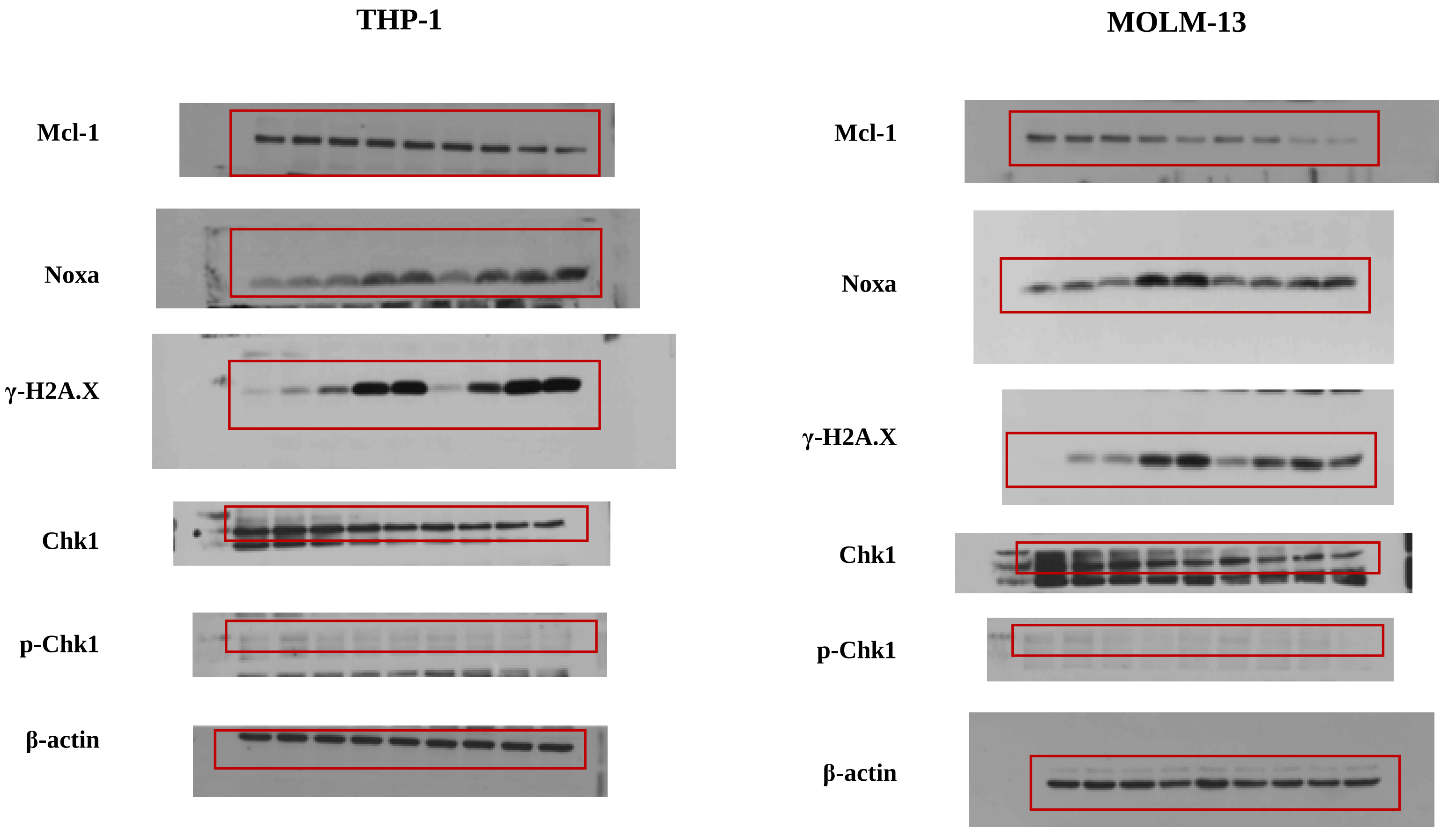
THP-1
MOLM-13
Mcl-1
Noxa
γ-H2A.X
Chk1
p-Chk1
β-actin
Mcl-1
Noxa
γ-H2A.X
Chk1
p-Chk1
β-actin

Fig. 5C

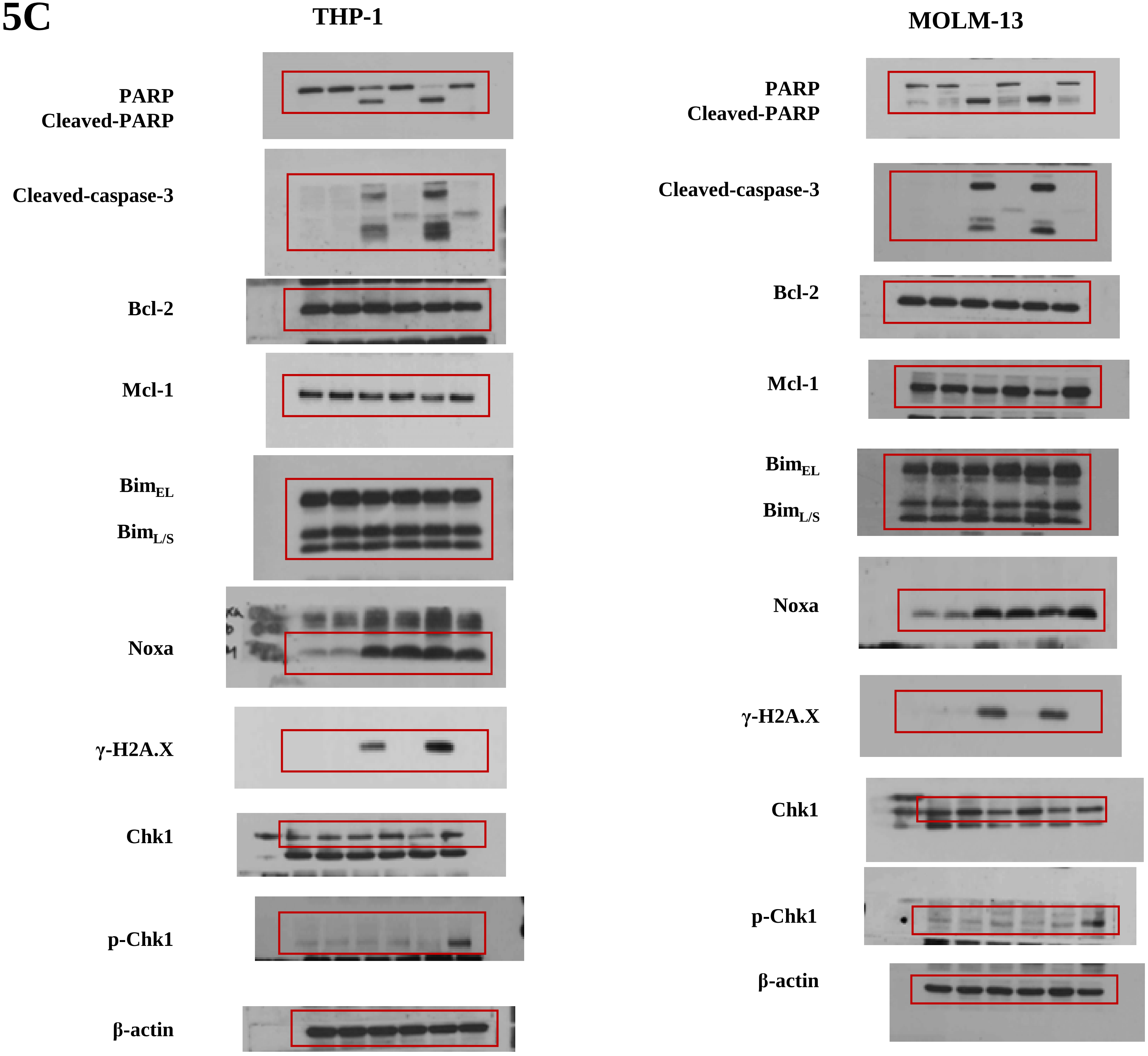
THP-1
MOLM-13
PARP
Cleaved-PARP
Cleaved-caspase-3
Bcl-2
Mcl-1
Bim_{EL}
$Bim_{L/S}$
Noxa
γ-H2A.X
Chk1
p-Chk1
β-actin
PARP
Cleaved-PARP
Cleaved-caspase-3
Bcl-2
Mcl-1
Bim_{EL}
$Bim_{L/S}$
Noxa
γ-H2A.X
Chk1
p-Chk1
β-actin

Fig. 6A

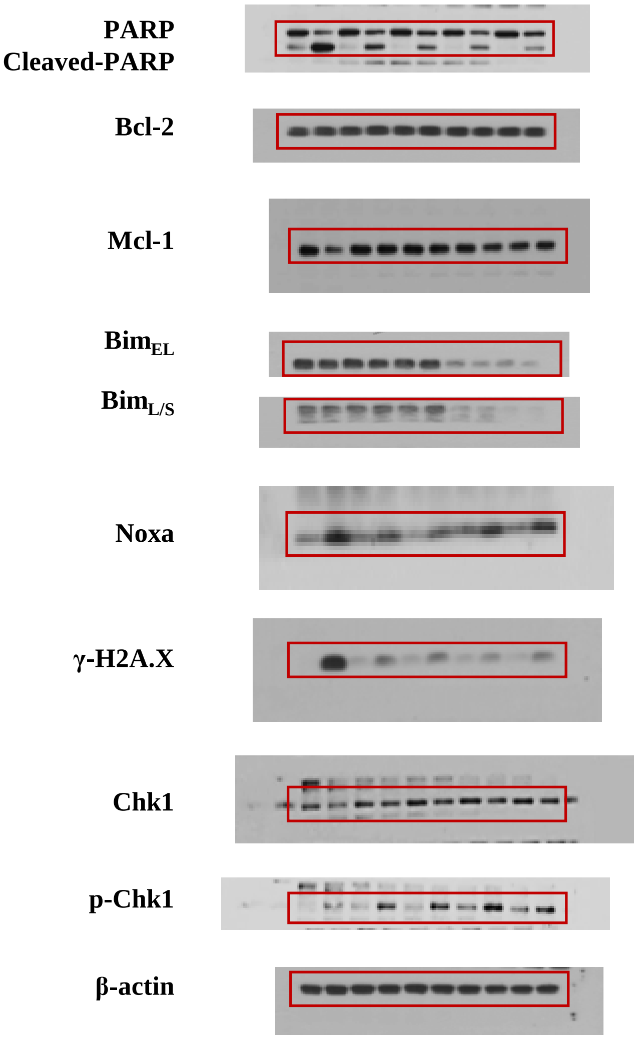

PARP
Cleaved-PARP
Bcl-2
Mcl-1
Bim_{EL}
$Bim_{L/S}$
Noxa
γ-H2A.X
Chk1
p-Chk1
β-actin

Fig. S3C

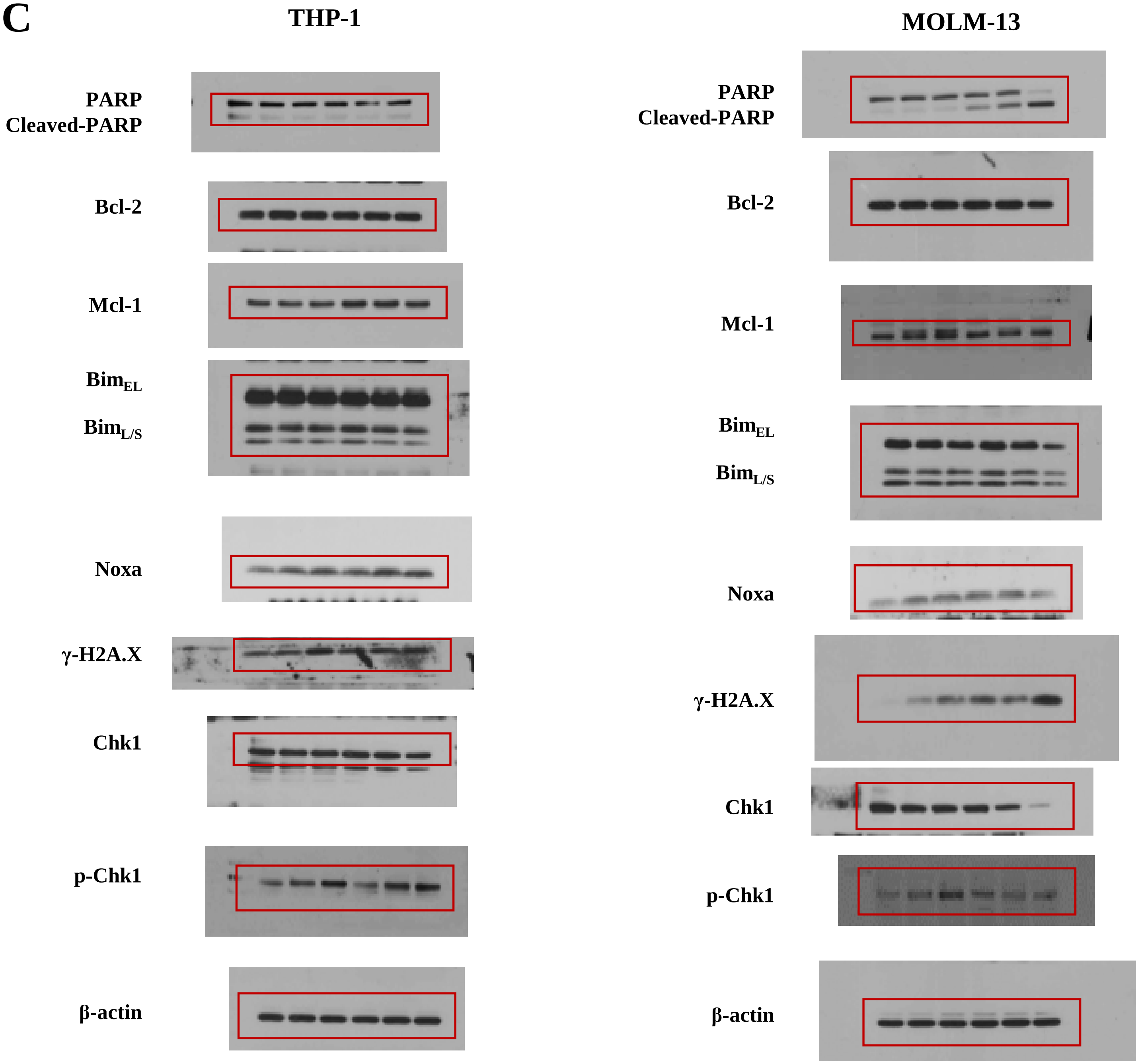
THP-1
MOLM-13
PARP
Cleaved-PARP
Bcl-2
Mcl-1
Bim_{EL}
$Bim_{L/S}$
Noxa
γ-H2A.X
Chk1
p-Chk1
β-actin
PARP
Cleaved-PARP
Bcl-2
Mcl-1
Bim_{EL}
$Bim_{L/S}$
Noxa
γ-H2A.X
Chk1
p-Chk1
β-actin
